# ﻿Two new species of *Dixonius* from Vietnam and Laos with a discussion of the taxonomy of *Dixonius* (Squamata, Gekkonidae)

**DOI:** 10.3897/zookeys.1163.101230

**Published:** 2023-05-23

**Authors:** Vinh Quang Luu, Thuong Huyen Nguyen, Minh Duc Le, Jesse L. Grismer, Hong Bich Ha, Saly Sitthivong, Tuoi Thi Hoang, L. Lee Grismer

**Affiliations:** 1 Faculty of Forest Resources and Environmental Management, Vietnam National University of Forestry, Xuan Mai, Chuong My, Hanoi, Vietnam; 2 Herpetology Laboratory, Department of Biology, La Sierra University, 4500 Riverwalk Parkway, Riverside, California 92505, USA; 3 Faculty of Environmental Sciences, University of Science, Vietnam National University, Hanoi, 334 Nguyen Trai Road, Hanoi, Vietnam; 4 Central Institute for Natural Resources and Environmental Studies, Vietnam National University, Hanoi, 19 Le Thanh Tong, Hanoi, Vietnam; 5 Department of Herpetology, American Museum of Natural History, Central Park West at 79; 6 th; 7 Street, New York, New York 10024, USA; 8 College of Forestry Biotechnology, Vietnam National University of Forestry, Hanoi, Vietnam; 9 Faculty of Forestry, National University of Laos, Dong Dok Campus, Vientiane, Lao PDR; 10 Department of Herpetology, San Diego Natural History Museum, PO Box 121390, San Diego, California, 92112, USA

**Keywords:** Gekkota, Indochina, integrative taxonomy, molecular phylogeny, morphology, new species, Southeast Asia

## Abstract

Integrated analyses using maximum likelihood (ML), Bayesian inference (BI), principal component analysis (PCA), discriminate analysis of principal components (DAPC), multiple factor analysis (MFA), and analysis of variance (ANOVA) recovered two new diagnosable species of gekkonid lizards in the genus *Dixonius*, one from the Central Highlands, Gia Lai Province, Vietnam and another from the Vientiane Province, Laos. Phylogenetic analyses based on the mitochondrial NADH dehydrogenase subunit 2 gene (ND2) and adjacent tRNAs showed that *Dixoniusgialaiensis***sp. nov.** is the sister species of *D.minhlei* from Dong Nai Province, Vietnam and is nested within a clade that also includes the sister species *D.siamensis* and *D.somchanhae*. *Dixoniusmuangfuangensis***sp. nov.** is the sister species to *D.lao* from Khammouane Province, Laos and is embedded in a clade with *D.vietnamensis*, *D.taoi*, and undescribed species from Thailand. Multivariate (PCA, DAPC, and MFA) and univariate (ANOVA) analyses using combinations of 15 meristic (scale counts), six morphometric (measurements), and five categorical (color pattern and morphology) characters from 44 specimens encompassing all eight species of *Dixonius* from Vietnam and Laos clearly illustrate *Dixoniusgialaiensis***sp. nov.** and *Dixoniusmuangfuangensis***sp. nov.** are statistically different and discretely diagnosable from all closely related species of *Dixonius*. These integrative analyses also highlight additional taxonomic issues that remain unresolved within *Dixonius* and the need for additional studies. The discovery of these new species further emphasizes the underappreciated herpetological diversity of the genus *Dixonius* and illustrates the continued need for field work in these regions.

## ﻿Introduction

**Figure 1.**
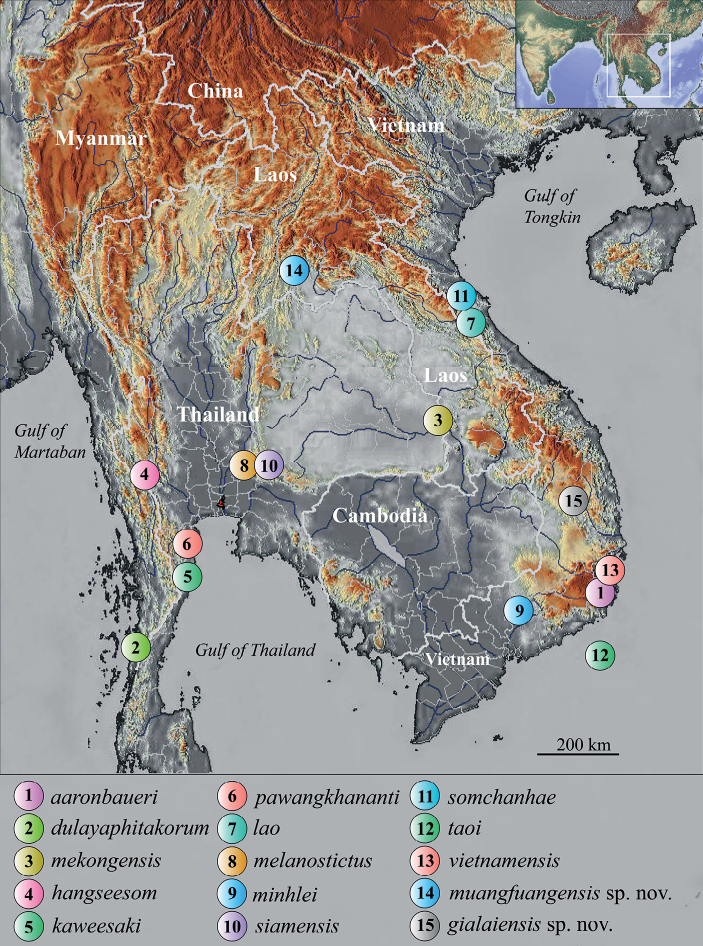
Location of the type localities of all known species of *Dixonius*. The inset delimits the study area. **1***Dixoniusaaronbaueri* from Ninh Thuan Province, Vietnam; **2***D.dulayaphitakorum* from Ranong Province, Thailand; **3***D.mekongensis* from Ubon Ratchathani Province, Thailand; **4***D.hangseesom* from Kanchanaburi Province, Thailand; **5***D.kaweesaki* from Prachuap Khiri Khan Province, Thailand; **6***D.pawangkhananti* from Phetchaburi Province, Thailand; **7***D.lao* from Khammouane Province, Laos; **8***D.melanostictus* from Nakhon Ratchasima Province, Thailand; **9***D.minhlei* from Dong Nai Province, Vietnam; **10***D.siamensis* from SaraBuri and Nakhon Ratchasima provinces, Thailand; **11***D.somchanhae* from Vientiane Capital, Laos; **12***D.taoi* from Binh Thuan Province, Vietnam; **13***D.vietnamensis* from Khanh Hoa Province, Vietnam; **14***D.muangfuangensis* sp. nov. from Vientiane Province, Laos; **15***D.gialaiensis* sp. nov. from Gia Lai Province, Vietnam.

The genus *Dixonius* was established by Bauer et al. (1997) to contain two species, *D.melanostictus* (Taylor, 1962) and *D.siamensis* (Boulenger, 1898), with a distribution range through Myanmar, Thailand, Laos, Vietnam, and Cambodia. Currently, thirteen species have been recognized worldwide (Nguyen et al. 2020, 2021; Pauwels et al. 2021; Uetz et al. 2022). In Vietnam, six species of *Dixonius* have been documented, including four originally described from the country, i.e., *D.vietnamensis* (Das 2004) from Khanh Hoa and Binh Thuan provinces, *D.aaronbaueri* (Ngo and Ziegler 2009) from Ninh Thuan and Binh Thuan provinces, *D.taoi* (Botov, Phung, Nguyen, Bauer, Brennan & Ziegler, 2015) from Binh Thuan Province, *D.minhlei* (Ziegler, Botov, Nguyen, Bauer, Brennan, Ngo & Nguyen, 2016) from Dong Nai Province, and two from outside Vietnam, *D.siamensis* from Thailand and Cambodia and *D.melanostictus* from Thailand (Uetz et al. 2022). Lastly, in Laos, there are three species (*D.siamensis*, *D.lao* (Nguyen, Sitthivong, Ngo, Luu, Nguyen, Le & Ziegler, 2020), *D.somchanhae* (Nguyen, Luu, Sitthivong, Ngo, Nguyen, Le & Ziegler, 2021)) two of which, *D.lao* from Vientiane Capital and *D.somchanhae* from Khammouane Province, were described within the last five years (Fig. 1).

During a recent herpetofaunal surveys in Chu Se Mountain Pass, Hbong Commune, Gia Lai Province in Vietnam and Vientiane Province in Laos, new populations of *Dixonius* were found at each location (Fig. 1). Based on phylogenetic evidence from the mitochondrial NADH dehydrogenase subunit 2 (ND2) gene and adjacent tRNAs, morphometric, meristic, and color pattern data, neither can be ascribed to any known species and as such they are described below as new species.

## ﻿Materials and methods

A total of six *Dixonius* specimens were caught by hand from Gia Lai Province, Vietnam and Vientiane Province, Laos. The specimens were fixed in approximately 80% ethanol and then transferred to 70% ethanol for permanent storage. Tissue samples taken before the specimens were preserved were stored separately in 95% ethanol. The specimens have been deposited in the collection of the Vietnam National University of Forestry (**VNUF**), Hanoi, Vietnam and the National University of Laos (**NUOL**), Vientiane, Laos.

### ﻿Species delimitation

The general lineage concept (GLC: de Queiroz 2007) adopted herein proposes that a species constitutes a population of organisms evolving independently from other such populations owing to a lack of, or limited gene flow. By “independently,” it is meant that new mutations arising in one species cannot spread readily into another species (Barraclough et al. 2003; de Queiroz 2007). Molecular phylogenies recovered multiple monophyletic mitochondrial lineages of individuals (populations) that were used to develop initial species-level hypotheses, the grouping stage of Hillis (2019). Discrete color pattern data and univariate and multivariate analyses of morphological data were then used to search for characters and morphospatial patterns consistent with the tree-designated species-level hypotheses, the construction of boundaries representing the hypothesis-testing step of Hillis (2019), thus providing independent diagnoses to complement the molecular analyses. In this way, delimiting (phylogeny) and diagnosing (taxonomy) species are not conflated (Frost and Hillis 1990; Frost and Kluge 1994; Hillis 2019).

### ﻿Molecular data and phylogenetic analyses

Four samples of the newly collected specimens were analyzed, two from Gia Lai Province, Vietnam (VNUF R.2020.22 – field number GL.02, VNUF R.2020.33 – field number GL.03) and two from Vientiane Province, Laos (VNUF R.2022.42 – field number MF.02, VNUF R.2022.52 – field number MF.03). We used the protocols of Nguyen et al. (2021) for DNA extraction, amplification, and sequencing. The complete NADH dehydrogenase subunit 2 (ND2) gene with six partial or complete adjacent tRNAs, approximately 1200 bp long, respectively, were amplified and sequenced using the primer pair, MetF1(5’-AAGCTTTCGGGCCCATACC-3’) and COIR1(5’-AGRGTGCCAATGTCTTTGTGRTT-3’) (Macey et al. 1997). Genomic DNA was extracted from all liver tissues stored in ethanol following the standard protocols of DNeasy blood and tissue kit, Qiagen (California, USA). The PCR volume consisted of 20 µl (1 µl each primer, 7 µl water, 10 µl of Taq mastermix and 1 µl DNA template). PCR conditions were: 95 °C for 5 min, followed by 42 cycles: 95 °C for 30 s, 50 °C for 45 s and 72 °C for 60 s with a final elongation step for 6 min at 72 °C. PCR products were visualized using electrophoresis through a 1.2% agarose gel, marker 100 bp, 1X TAE and stained with RedSafe Nucleic Acid Staining Solution and photographed under UV light of Geldoc system (Quantum CX5, Villber, France). Successful amplifications were purified using innuPREP Gel Extraction Kit (Analytik Jena, Germany). Cleaned PCR products were sent to 1^st^ Base (Malaysia) for sequencing in both directions.

We obtained 1,444 base pairs of NADH dehydrogenase subunit 2 gene (ND2) sequence data and the flanking tRNAs from 29 ingroup individuals of *Dixonius* representing 13 nominal species including the new samples from Vietnam and Laos. *Heteronotiaspelea* was used as an outgroup to root the tree based on the phylogenetic results generated by Gamble et al. (2015). Sequence data for other species were acquired from GenBank. Newly generated sequences were deposited in GenBank (Table 1).

**Table 1.** List of specimens used for the phylogenetic analyses.

Species	Catalog no.	Location	GenBank no.
*Dixoniusaaronbaueri*	ZFMK87274	Nui Chua NP, Ninh Thuan Province, southern Vietnam	HM997152
*Dixoniusgialaiensis* sp. nov.	VNUF R.2020.22 (Field no. GL.02)	Chu Se District, Gia Lai Province, Vietnam	OQ819041
	VNUF R.2020.33 (Field no. GL.03)	Chu Se District, Gia Lai Province, Vietnam	OQ8190412
*Dixoniuslao*	VNUF R.2016.2	Khammouane Province, Laos	MT024681
	IEBR A.2019.5	Khammouane Province, Laos	MT024683
	IEBR A.2019.6	Khammouane Province, Laos	MT024682
*Dixoniusmelanostictus*	VU 022	Captive, Thailand	HM997153
*Dixoniusminhlei*	ZFMK 97745	Vinh Cuu, Dong Nai Province, Vietnam	KX379194
*Dixoniusmuangfuangensis* sp. nov.	VNUF R.2020.42 (Field no. MF02)	Muangfuang District, Vientiane Province, Central Laos	OQ818586
	VNUF R.2020.52 (Field no. MF03)	Muangfuang District, Vientiane Province, Central Laos	OQ818587
Dixoniuscf.siamensis	VU 023	Captive, Thailand	KX379195
*Dixoniussiamensis*	LSUHC 7328	Phnom Aural, Purset Province, Cambodia	EU054299
	FMNH 263003	Keo Seima District, Mondolkiri- Province, Cambodia	EU054298
	LSUHC 7378	Phnom Aural, Purset Province, Cambodia	KP979732
*Dixoniussomchanhae*	VNUF R.2020.2	Nasaithong District, Vientiane Capital, Laos	MW605166
	VNUF R.2020.1	Nasaithong District, Vientiane Capital, Laos	MW605165
	VNUF R.2020.3	Nasaithong District, Vientiane Capital, Laos	MW605167
	VNUF R.2020.55 (Field no. VT05)	Vientiane Capital, Laos	OQ818589
	VNUF R.2020.54 (Field no. VT04)	Vientiane Capital, Laos	OQ818588
	VNUF R.2020.59 (Field no.VT09)	Vientiane Capital, Laos	OQ818591
	VNUF R.2020.56 (Field no. VT0T06)	Vientiane Capital, Laos	OQ818590
*Dixonius* sp.	LSUHC 9466	Sai Yok, Kanchanaburi Province, Thailand	KX379196
*Dixoniustaoi*	ZFMK 96680	Phu Quy Island, Binh Thuan Province, Vietnam	KP979733
	CAS 257300	Phu Quy Island, Binh Thuan Province, Vietnam	KP979734
	IEBR A 2014-26	Phu Quy Island, Binh Thuan Province, Vietnam	KP979735
	IEBR A 2014-27	Phu Quy Island, Binh Thuan Province, Vietnam	KP979736
Dixoniuscf.vietnamensis	ZFMK 87273	Nui Chua, Ninh Thuan Province, Vietnam	KX379201
*Dixoniusvietnamensis*	IEBR R.20163	Nha Trang, Khánh Hòa Province, Vietnam	KX379198

Maximum likelihood (ML) and Bayesian inference (BI) were used to estimate phylogenetic trees. Best-fit models of evolution determined in IQ-TREE (Nguyen et al. 2015) using the Bayesian information criterion (BIC) implemented in ModelFinder (Kalyaanamoorthy et al. 2017) indicated that F81+F was the best-fit model of evolution for the tRNAMET and K2P+I, and HKY+F+G4 were the best models of evolution for tRNAs2 and ND2, respectively. The ML analysis was performed using the IQ-TREE webserver (Trifinopoulos et al. 2016) with 1000 bootstrap pseudoreplicates using the ultrafast bootstrap (UFB) analysis (Minh et al. 2013; Hoang et al. 2018). The BI analysis was performed on CIPRES Science Gateway (Miller et al. 2010) using MrBayes v. 3.2.4 (Ronquist et al. 2012). Two independent runs were performed using Metropolis-coupled Markov Chain Monte Carlo (MCMCMC), each with four chains: three hot and one cold. The MCMCMC chains were run for 80,000,000 generations with the cold chain sampled every 8000 generations and the first 10% of each run being discarded as burn-in. The posterior distribution of trees from each run was summarized using the sumt function in MrBayes v. 3.2.4 (Ronquist et al. 2012). Stationarity was checked with Tracer v. 1.6 (Rambaut et al. 2014) to ensure effective sample sizes (ESS) for all parameters were well above 200. We considered Bayesian posterior probabilities (BPP) of 0.95 and above and ultrafast bootstrap support values (UFB) of 95 and above as an indication of strong nodal support (Huelsenbeck et al. 2001; Minh et al. 2013). Uncorrected pairwise sequence divergences (p-distance) were calculated in MEGA 11 (Kumar et al. 2016) using the complete deletion option to remove gaps and missing data from the alignment prior to analysis.

A time-calibrated Bayesian phylogenetic tree was estimated using BEAST 2 (Bayesian Evolutionary Analysis by Sampling Trees) v. 2.7.3 (Drummond et al. 2012) implemented in CIPRES (Cyberinfrastructure for Phylogenetic Research; Miller et al. 2010) where the ingroup node subtending the split between *Dixoniusaaronbaueri* and the remaining species was given a 24.04 mya prior with an offset range of 20.23–27.68 mya following Gamble et al. (2015). The split between *Heteronotia* and *Dixonius* was set at 45.0 mya with an offset range of 33.3–56.8 mya (Gamble et al. 2015). An input file was constructed in BEAUti (Bayesian Evolutionary Analysis Utility) v. 2.7.3. An optimized relaxed clock with unlinked site models, linked clock models and linked trees, and a calibrated Yule prior were employed for the species level. BEAST Model Test (Bouckaert and Drummond 2017), implemented in BEAST, was used to numerically integrate over the uncertainty of substitution models while simultaneously estimating phylogeny using Markov chain Monte Carlo (MCMC). MCMC chains were run for 80 million generations and logged every 8,000 generations. The BEAST log file was visualized in Tracer v. 1.7.2 (Rambaut et al. 2014) to ensure effective sample sizes (ESS) were above 200 for all parameters. A maximum clade credibility tree using mean heights at the nodes was generated using TreeAnnotator v. 2.7.3 (Rambaut and Drummond 2013) with a burn-in of the first 10% of each run. Nodes with Bayesian posterior probabilities (BPP) of 0.95 and above were considered strongly supported (Huelsenbeck et al. 2001; Wilcox et al. 2002).

### ﻿Morphological data and analysis

The morphological data set comprised six closely related species including six type specimens of *Dixoniusminhlei* from Dong Nai Province, Vietnam (IEBR A.0801-02, VNMN R.2016.1-2, ZFMK 97745-46), three type specimens of *D.lao* from Khammouane Province, Laos (VNUF R.2016.2, IEBR A.2016.5-6), eight specimens of *D.siamensis* from Pursat Province, Cambodia (LSUHC 07328, 07378, 08420, 08487, 08491, 08522, 09284, 09289), five type specimens of *D.somchanhae* from Vientiane Capital, Laos (VNUF R.2020.1-5), four specimens of *D.* sp. from Gia Lai Province, Vietnam, and 12 specimens of *D.vietnamensis* from Nha Trang Province, Vietnam (ZRC 2.6024-27, IEBR R.2016.1, 2016.3, 2016.4, VNMN R.2016.3-4, ZFMK 97747-49).

Morphological data included both meristic and morphometric characters. Morphological characters were taken from the 44 specimens following Bauer et al. (2004) and Ngo and Ziegler (2009). Morphometric characters were taken after preservation with a digital caliper to the nearest 0.1 mm under a zoom stereomicroscope on the right/left of the body. Recorded data included: **SVL**: snout-vent length (taken from the tip of the snout to the vent), **TaL**: tail length (taken from the vent to the tip of the tail, original or partially regenerated), **TW**: tail width (taken at the base of the tail immediately posterior to the postcloacal swelling), **BW**: body width (greatest width of torso, taken at the level of midbody), **HL**: head length (the distance from the posterior margin of the retroarticular process of the lower jaw to the tip of the snout), HW: head width (the distance from the posterior margin of the retroarticular process of the lower jaw to the tip of the snout), **HD**: head depth (the maximum height of head measured from the occiput to base of the lower jaw), **EL**: ear length (greatest oblique length across the auditory meatus), **TBL**: Tibia length (taken on the ventral surface from the posterior surface of the knee while flexed 90° to the base of the heel), **AG**: axilla to groin length (taken from the posterior margin of the forelimb at its insertion point on the body to the anterior margin of the hind limb at its insertion point on the body), **FA**: forearm length (taken on the ventral surface from the posterior margin of the elbow while flexed 90° to the inflection of the flexed wrist), **ED**: eye diameter (the greatest horizontal diameter of the eye-ball), **EN**: eye nostril distance (measured from the anterior margin of the bony orbit to the posterior margin of the external nares), **ES**: eye snout distance (measured from anteriormost margin of the bony orbit to the tip of snout), **EE**: eye ear distance (measured from the anterior edge of the ear opening to the posterior edge of the bony orbit), **IN**: internarial distance (measured between the external nares across the rostrum), **IO**: interorbital distance (measured between the dorsal-most edges of the bony orbits).

Meristic data taken were: **V**: ventral scales (counted transversely across the abdomen midway between limb insertions from one ventrolateral fold to the other), **DTR**: longitudinal rows of dorsal tubercles (counted transversely across the body midway between the limb insertions from one ventrolateral body fold to the other), **PV**: paravertebral scales (counted in a paravertebral row from first scale posterior to parietal scale to last scale at the level of vent opening), **PV`**: paravertebral scales (counted in a row between limb insertions), **T4**: lamellae under fourth toe (counted from the distal scale containing claw to basal scale that broadly contacts adjacent fragmented scales), **IOS**: Interorbital scales (counted at narrowest point between orbits), **ICS**: interciliary scales (counted between supraciliaries at midpoint of orbit), **SPL**: supralabials (counted from the largest scale at the corner of the mouth to the rostral scale), **IFL**: infralabials (counted from termination of enlarged scales at the corner of the mouth to the mental scale), **MO**: number of supralabial at midorbital position, **PP**: precloacal pores in males.

Color pattern on dorsum including the presence or absence of canthal stripes (**CanthStrp**), the presence or absence of strong darkly barred lips (**LipBar**), the presence or absence of dark-colored round blotches on the top of the head (**RdHdBlch**) and dorsum (RdBodBlch), and the presence or absence of two regularly arranged whitish tubercles on flanks (**Tub**). The raw morphological data for all characters and specimens are presented in Tables 2, 3.

**Table 2.** Sex and raw meristic and categorical data used in the analyses from specimens of *Dixonius* from Vietnam and Laos. m = male; f = female; j = juvenile; r/l = right/left.

Species	Museum no.	Sex	Meristic data						Categorical data				
			SPL r/l	IFL r/l	MO	IOS	V	T4 r/l	Canthal stripe	Lips strong barred	Blotches on the head round	Blotches on dorsum round	Two regularly disposed whitish tubercles on each side of the flanks
*minhlei*	IEBR A.0802	m	8	6	6	10	22	14	present	no	yes	yes	absent
	ZFMK 97746	m	8	6.5	6	10	23	14.5	present	no	yes	yes	absent
	IEBR A.0801	f	8.5	7	6	10	22	12	present	no	yes	yes	absent
	ZFMK 97745	f	7.5	6	5.5	10	23	13	present	no	yes	yes	absent
	VNMN R.2016.1	f	8	6	5.5	8	23	15	present	no	yes	yes	absent
	VNMN R.2016.2	f	8	6.5	6	7	20	13	present	no	yes	yes	absent
*gialaiensis* sp. nov.	VNUF R.2020.22	m	7.5	6	6	7	21	14	present	yes	yes	yes	present
	VNUF R.2020.33	f	7	6	6	7	19	14	present	yes	yes	yes	present
	VNUF R.2020.44	mj	8	7	6	7	21	14.5	present	yes	yes	yes	present
*vietnamensis*	ZRC 2.6024	m	5	6	5	10	20	13	present	no	no	no	present
	ZRC 2.6025	m	5	6	5	9	20	13	present	no	no	no	present
	ZRC 2.6026	j	5	6	6	8	20	13	present	no	no	no	present
	ZRC 2.6027	j	6	7	6	8	20	13	present	no	no	no	present
	IEBR R.2016.3	m	8	6	5.5	10	19	13.5	present	no	no	no	present
	VNMN R.2016.3	m	7.5	6	5.5	9	19	13.5	present	no	no	no	present
	IEBR R.2016.1	f	7	6	5.5	8	18	13.5	present	no	no	no	present
	VNMN R.2016.4	f	7.5	7	6	9	20	13	present	no	no	no	present
	ZFMK 97748	f	7.5	6	6	8	20	14	present	no	no	no	present
	ZFMK 97747	mj	7.5	6	5.5	10	15	13.5	present	no	no	no	present
	IEBR R.2016.4	f j	8	7	6	7	21	12.5	present	no	no	no	present
	ZFMK 97749	fj	7	6.5	5.5	8	19	13.5	present	no	no	no	present
sp.	VNUF R.2022.81	m	8	6.5	6	9	24	14	present	no	no	yes	present
	VNUF R.2022.82	f	7.5	5.5	6	8	23	14.5	present	no	no	yes	present
	VNUF R.2022.83	fj	8	7	6	8	23	14	present	no	no	yes	present
	VNUF R.2022.84	f j	8.5	6	6	8	22	13.5	present	no	no	yes	present
*somchanhae*	VNUF R.2020.3	m	7	5	6	8	24	14	present	yes	no	no	present
	VNUF R.2020.2	m	8	6	6	8	23	15	present	yes	no	no	present
	VNUF R.2020.1	m	8	5.5	6	8	23	15	present	yes	no	no	present
	VNUF R.2020.4	f	8	5.5	6	8	23	15	present	yes	no	no	present
	VNUF R.2020.5	f	8	6	6	7	26	13	present	yes	no	no	present
*siamensis*	LSUHC09284	f	8	7	6	9	19	14	absent	yes	no	yes	present
	LSUHC08522	f	8	6.5	6	10	22	14.5	absent	yes	no	yes	present
	LSUHC08487	f	8	7	6	10	20	14.5	absent	yes	no	yes	present
	LSUHC08420	m	8.5	7	6	10	21	13	absent	yes	no	yes	present
	LSUHC08491	f	8	7	6	9	20	14.5	absent	yes	no	yes	present
	LSUHC07328	j	7.5	6	5.5	9	22	14	absent	yes	no	yes	present
	LSUHC07378	m	8	6	6	10	20	14.5	absent	yes	no	yes	present
	LSUHC09289	m	7.5	6	6	10	21	16	absent	yes	no	yes	present
*muangfuangensis* sp. nov.	NUOL R.2022.01	m	7	6.5	6	7	21	15	absent	yes	no	no	present
	VNUF R.2020.42	m	8	7	6	7	20	15	absent	yes	no	no	present
	VNUF R.2020.52	f	8	6.5	6	7	21	15	absent	yes	no	no	present
*lao*	VNUF R.2016.2	m	9.5	8	7.5	9	23	15	absent	yes	no	no	absent
	IEBR A.2019.5	f	8.5	8	7	8	23	15	absent	yes	no	no	absent
	IEBR A.2019.6	f	9	7.5	8	8	24	15	absent	yes	no	no	absent

**Table 3.** Sex and raw morphometric data used in the analyses from specimens of *Dixonius* from Vietnam and Laos. m = male; f = female; j = juvenile.

Species	Museum no.	Sex	SVL	BW	HL	HW	HD	EL	ED	EN	ES	EE	IN	IO	FAr	TBLr	AGr
*minhlei*	IEBR A.0802	m	43.9	9.4	7.3	7.7	4.7	1.5	2.7	3.7	5	3.5	1.6	4	6.2	7.7	18.7
	ZFMK 97746	m	40.6	8.5	6.7	6	4.3	1.3	2.2	3.2	4.4	3.6	1.3	3.5	6.7	7	18.2
	IEBR A.0801	f	45.9	9.7	7.2	6.6	5.2	1.2	3.3	3.4	4.4	3.4	1.5	3.7	5.9	7.2	21.2
	ZFMK 97745	f	47.5	9.6	7.6	6.8	4.7	1.5	3.1	3.5	4.9	3.9	1.5	3.7	6	7.3	21.5
	VNMN R.2016.1	f	43.3	9.3	7.1	6.5	4.4	1.3	2.5	3.5	4.6	3.8	1.5	3.8	6.1	7.5	20.6
	VNMN R.2016.2	f	46.7	9.2	7.7	6.2	4.6	1.2	3.1	3.8	5.2	3.6	1.5	3.4	6.6	7	30.3
*gialaiensis* sp. nov.	VNUF R.2020.22	m	41.2	8.6	11.7	7.7	5.2	1.1	2.9	3.1	4.3	3.3	1.3	1.2	6.1	6.9	15.8
	VNUF R.2020.33	f	47.4	8.4	12.3	8.8	6.1	1.3	3.3	3.5	4.8	3.5	1.5	1.4	6.3	7.7	21.8
	VNUF R.2020.44	mj	35.9	8.3	10.9	6.8	4.7	0.9	2.6	2.9	3.8	3	1.3	1.3	4.5	5.6	14
*vietnamensis*	ZRC 2.6024	m	40.8	8	7.5	7.9	5.5	1	2.9	3.2	4.3	3.8	2.1	3.6	5.6	7.7	21
	ZRC 2.6025	m	42.4	9.1	7.5	7.6	6	1.1	2.9	3.7	4.6	4	1.6	3.6	6.2	7.2	21
	ZRC 2.6026	j	26.6	5.4	5.4	5.2	4	0.6	2.1	2.2	3	2.5	1.1	2.7	4.4	4.4	13
	ZRC 2.6027	j	25.9	4	5.2	5.1	3.3	0.6	1.8	2.3	3.5	2.2	0.9	2.1	4	4.6	11.8
	IEBR R.2016.3	m	39	6.5	6.9	6.8	4.2	1.1	2.8	2.9	3.9	3.1	1.1	1.6	4.7	6.5	14.8
	VNMN R.2016.3	m	39.9	7.8	7.2	7	4.7	0.8	2.5	3.4	4.6	3.3	1.3	1.7	5.2	6.5	16.6
	IEBR R.2016.1	f	43.5	7.6	7.6	6.9	4.7	1	2.7	3.1	4.5	2.7	1.3	1.6	5	6.2	19.2
	VNMN R.2016.4	f	43.7	8.6	7.7	7.7	4.7	1.1	2.8	3.4	4.7	3.6	1.3	1.8	5.5	6.3	18.2
	ZFMK 97748	f	45.2	10.3	8.5	8.2	5.7	1.3	2.9	3.8	5.4	4.3	1.5	2.7	5.5	6.6	19.2
	ZFMK 97747	mj	34.1	4.7	6.2	5.7	4.1	0.9	2.3	2.7	3.6	2.5	1.2	1.3	4.2	5.9	12.3
	IEBR R.2016.4	f j	31.2	5.2	5.7	5.8	3.7	0.9	2.4	2.4	3.4	2.4	1	1.2	3.1	4.8	11.9
	ZFMK 97749	fj	29.2	4.8	5.2	4.8	3.1	0.9	2.5	2.1	2.9	2.3	1	1.2	3.1	4.9	11.1
sp.	VNUF R.2022.81	m	46	10.7	13.7	7.8	5.7	1.4	2.2	3.4	4.8	3.7	1.7	1.9	5.5	6.8	20.2
	VNUF R.2022.82	f	35.2	7.5	10.9	6.9	3.3	1.1	2.1	2.9	4.1	3.2	1.3	1.6	4.7	6.2	14.2
	VNUF R.2022.83	fj	31.1	5.1	9.2	5.1	2.4	1.2	2.2	2.3	2.3	2.5	1.1	1.4	4.5	5.1	12.5
	VNUF R.2022.84	f j	30.3	5.6	9.3	5.8	3	0.8	2	2.7	3.4	2.9	1.1	1.5	3.7	4.6	13.3
*somchanhae*	VNUF R.2020.3	m	43.8	9.4	12.2	8.5	5.6	1.6	3.4	3	5.1	3.4	1.3	1.7	6.2	7.3	20.5
	VNUF R.2020.2	m	47.1	11.1	12.9	9.7	5.9	1.9	3.3	3.4	5	3.5	1.6	1.8	5.6	8	19.5
	VNUF R.2020.1	m	39.8	8.9	11.6	7.9	5.2	1.2	2.9	2.9	4.2	3.1	1.7	1.3	4.8	6.6	17.4
	VNUF R.2020.4	f	35.5	9.4	9.7	6.9	4.2	1.2	2.2	2.8	3.7	2.5	1.2	1.4	4.3	5.5	15.5
	VNUF R.2020.5	f	39.9	8.9	11.4	7.6	4.4	1.5	3.1	2.6	4	3.1	1.5	1.3	4.9	6	19.7
*siamensis*	LSUHC09284	f	45.4	8.6	12.8	8.7	5.2	1.6	3	3.7	5.1	4	2	3.6	6.7	7.3	19
	LSUHC08522	f	44.1	9.4	12.5	8.1	5.7	1.4	2.4	4.4	5.2	4.5	1.8	3.7	6.7	6.9	21.2
	LSUHC08487	f	48.6	10.7	14.3	8.7	5.4	1.6	3.2	3.4	5.4	4.2	1.7	3.5	7.1	8	21.8
*siamensis*	LSUHC08420	m	46.9	8.8	13.1	9.1	5.3	1.3	2.7	3.7	5.3	3.9	1.5	3.7	6.7	7.3	20.7
	LSUHC08491	f	45.2	10.2	13	8.2	5.7	1.4	2.8	3.3	4.7	4.2	2	3.7	6.2	6.9	19
	LSUHC07328	j	28.6	5.8	8.4	5.5	3	0.7	2.1	2.4	3.3	2.8	1.5	2.8	3.8	5	12
	LSUHC07378	m	36.7	6.5	10.9	7.3	4.5	1.3	2.6	3.1	4.6	3.4	1.6	3.4	6	6.6	16.1
	LSUHC09289	m	45.3	9.1	12.7	8.6	5.1	1.6	2.6	3.7	5	3.6	2	3.5	7	7.3	18.9
*muangfuangensis* sp. nov.	NUOL R.2022.01	m	38.3	7.83	10.5	7.2	4.3	0.8	2.4	2.8	3	3.4	1.3	1.7	4.3	4.9	16.5
	VNUF R.2020.42	m	55.6	11.93	15.2	10.8	6.9	2.3	3	3.8	5.9	5.1	1.6	2.3	6.8	7.2	23.1
	VNUF R.2020.52	f	56.7	12.23	16.7	10.7	6.9	2.1	3.5	3.8	5.8	5.1	1.7	2.4	7.1	7.3	27.4
*lao*	VNUF R.2016.2	m	50.1	9.7	14.1	9.2	5.3	1.4	3.6	4.4	5.6	4.1	1.7	1.7	6.9	7.6	20.6
	IEBR A.2019.5	f	55.4	11.5	14.3	9.7	6.2	1.7	3.6	4	5.5	4.4	1.8	1.5	7.1	8.5	22.2
	IEBR A.2019.6	f	35.8	7.2	9.9	7	4	1.1	2.7	2.8	3.6	2.6	1.1	1.1	4.6	5.9	15.2

All statistical analyses were performed using R v. 4.2.1 (R Core Team, 2021). Morphometric characters used in the statistical analyses were SVL, BW, HL, HW, HD, EL, ED, EN, ES, EE, IN, IO, FAr, TBLr, and AGr. Tail metrics were not used due to the high degree incomplete sampling (i.e., regenerated, broken, or missing). To remove potential effects of allometry on morphometric traits (sec. Chan and Grismer 2022), we used the following equation: Xadj = log(X) – β[log(SVL) – log(SVLmean)], where Xadj = adjusted value; X = measured value; β = unstandardized regression coefficient for each population; and SVLmean = overall average SVL of all populations (Thorpe 1975, 1983; Turan 1999; Lleonart et al. 2000, accessible in the R package GroupStruct (available at https://github.com/chankinonn/ GroupStruct). The morphometrics of each species were normalized separately and then concatenated into a single data set so as not to conflate potential intra- with interspecific variation (Reist 1986; McCoy et al. 2006). All data were scaled to their standard deviation to ensure they were analyzed on the basis of correlation and not covariance. Meristic characters (scale counts) used in statistical analyses were SPLr/l, IFLr/l, MO, IOS, ICS, V, DTR, and T4r/l. Precloacal and femoral pores were omitted from the analyses due to their absence in females. Categorical characters analyzed were CanthStrp, LipBar, RdHdBlch, RdBodBlch, and Tub.

A Levene’s test for normalized morphometric and meristic characters was conducted to test for equal variances across all groups. Analyses of variance (ANOVA) were conducted on meristic and normalized morphometric characters (see below) with statistically similar variances to search for the presence of statistically significant mean differences (p < 0.05) among species across the data set. Characters bearing statistical differences were subjected to a TukeyHSD test to ascertain which species pairs differed significantly from each other for those particular characters. Boxplots were generated for discrete meristic characters in order to visualize the range, mean, median, and degree of differences between pairs of species bearing statistically different mean values and violin plots were generated for continuous morphometric characters to visualize the same.

Morphospatial positions were visualized using principal component analysis (PCA) from the ADEGENET package in R (Jombart et al. 2010) to determine if their positioning was consistent with the putative species boundaries delimited by the molecular phylogenetic analyses and defined by the univariate analyses (see above). PCA, implemented using the “prcomp()” command in R, is an indiscriminate analysis plotting the overall variation among individuals (i.e., data points) while treating each individual independently (i.e., not coercing data points into pre-defined groups). Subsequent to the PCA, a discriminant analysis of principle components (DAPC) was used to test for corroboration and further discrimination of morphospatial differences among the putative species. DAPC a priori groups the individuals of each predefined population inferred from the phylogeny into separate clusters (i.e., plots of points) bearing the smallest within-group variance that produce linear combinations of centroids having the greatest between-group variance (i.e., linear distance; Jombart et al. 2010). DAPC relies on standardized data from its own PCA as a prior step to ensure that variables analyzed are not correlated and number fewer than the sample size. Principal components with eigenvalues accounting for 90–95% of the variation in the data set were retained for the DAPC analysis according to the criterion of Jombart et al. (2010).

To test and further corroborate the PCA and DAPC analyses, we conducted a multiple factor analysis (MFA) on the above-mentioned morphological characters plus the categorical color pattern differences for a near total evidence data set (see Tables 5, 6). The MFA was implemented using the *mfa* () command in the R package FactorMineR (Husson et al. 2017) and visualized using the Factoextra package (Kassambara and Mundt 2017). MFA is a global, unsupervised, multivariate analysis that incorporates qualitative and quantitative data (Pagès 2015), making it possible to analyze different data types simultaneously in a nearly total evidence environment. In an MFA, each individual is described by a different set of variables (i.e., characters) which are structured into different data groups in a global data frame, in this case, quantitative data (i.e., meristics and normalized morphometrics) and categorical data (i.e., color pattern). In the first phase of the analysis, separate multivariate analyses are carried out for each set of variables, principal component analyses (PCA) for the quantitative data sets and multiple correspondence analysis (MCA) for categorical data. The data sets are then normalized separately by dividing all their elements by the square root of their first eigenvalues. For the second phase of the analysis, the normalized data sets are concatenated into a single matrix for a global PCA of the data. Standardizing the data in this manner prevents one data type from overleveraging another. In other words, the normalization of the data in the first phase prevents data types with the highest number of characters or the greatest amount of variation from outweighing other data types in the second phase. This way, the contribution of each data type to the overall variation in the data set is scaled to define the morphospatial distance between individuals as well as calculating each data type’s and each character’s contributions to the overall variation in the data set (Pagès 2015; Kassambara and Mundt 2017).

## ﻿Results

### ﻿Molecular results

**Figure 2.**
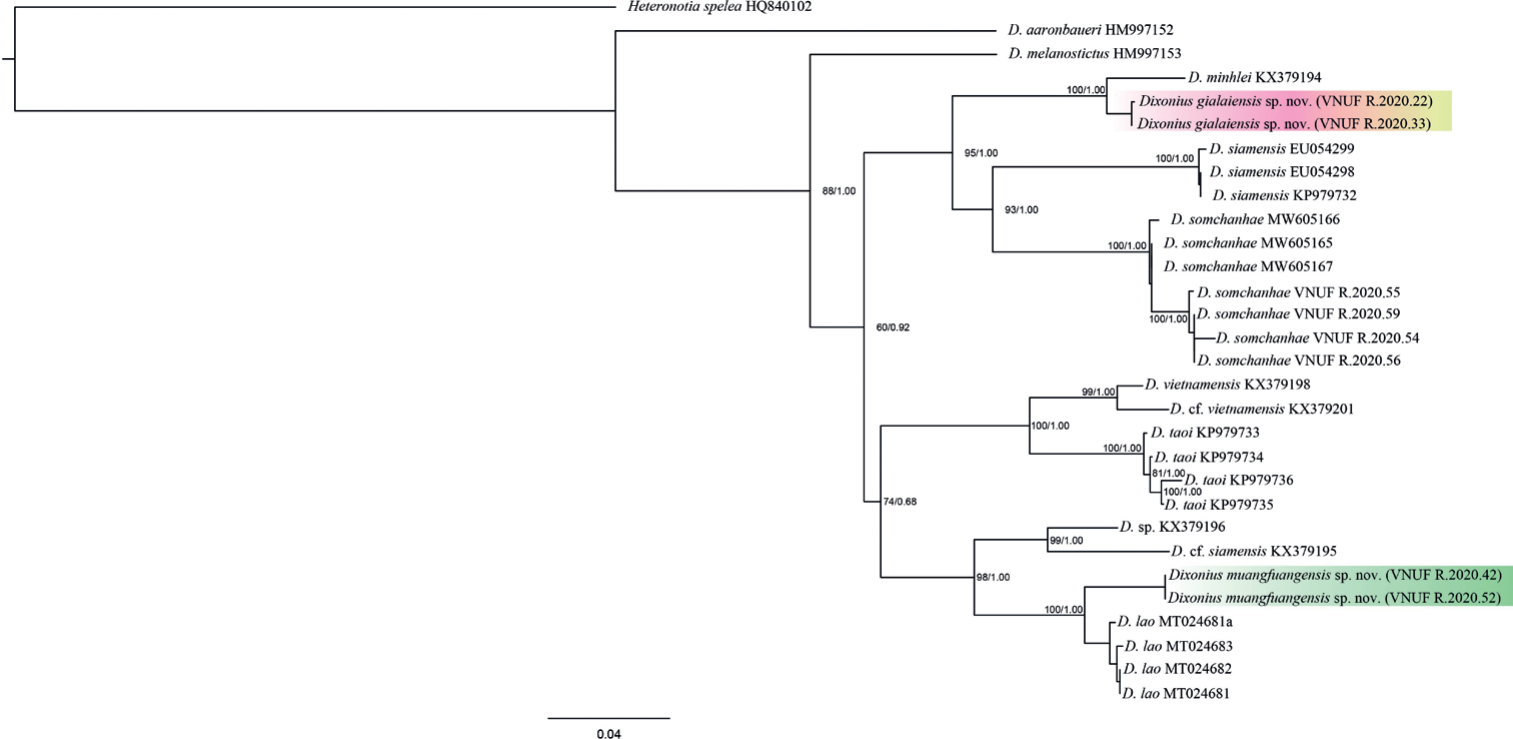
Maximum likelihood topology of the *Dixonius* species from Vietnam and Laos with ultrafast bootstrap values (UFB) and Bayesian posterior probabilities (BPP) at the nodes, respectively.

**Figure 3.**
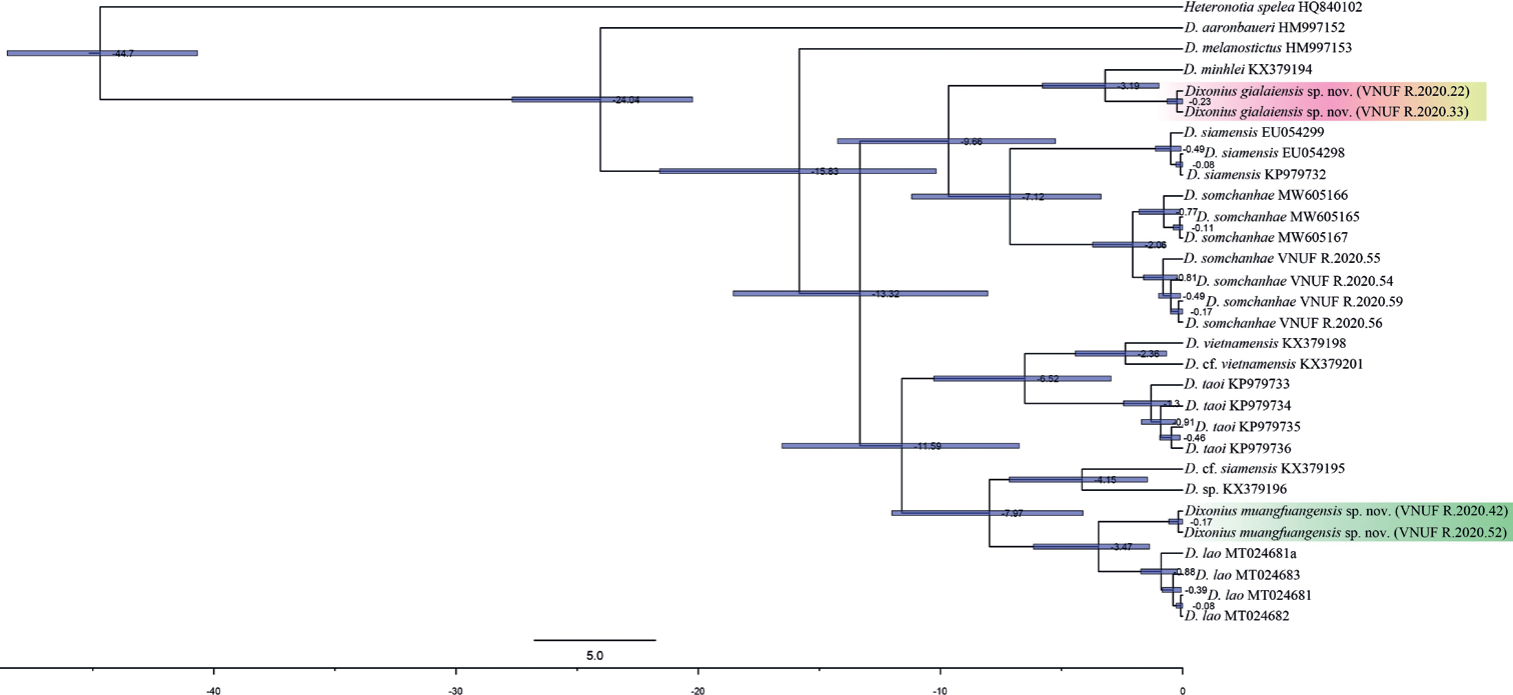
BEAST chronogram of the *Dixonius* species from Vietnam and Laos. Numbers at the nodes are mean ages in millions of years. Bars represent 95% highest posterior densities.

The results of ML, BI, and BEAST analyses produced trees with identical topologies and strong support at nearly every node (Figs 2, 3). The molecular analyses suggest that *Dixoniusaaronbaueri* is the sister species to a clade containing all other species of *Dixonius*. Additionally, all analyses recovered the newly discovered population from Chu Se District, Gia Lai Province, Vietnam as the strongly supported (1.00/100) sister species of *D.minhlei* and the newly discovered population from Muangfuang District, Vientiane Province, Laos as the strongly supported (1.00/100) sister species of *D.lao* (Figs 2, 3). Uncorrected pairwise sequence divergences among *Dixonius* species ranged from 2.57–18.84% (Table 4). Ranges for the new species described (see below) are as follows: new species from Vietnam 3.60–15.73%, being most similar to *D.minhlei* and most distant to *D.aaronbaueri* and new species from Laos 3.10–18.17%, being most similar to *D.lao* and most distant to *D.aaronbaueri*.

**Table 4.** Mean percentages of uncorrected pairwise sequence divergence (p-distances) among the species of *Dixonius*. Intraspecific p-distance are in bold font, n/a = data not applicable.

	*Dixonius* sp.	cf. siamensis	*aaronbaueri*	*taoi*	*vietnamensis*	cf. vietnamensis	*muangfuangensis* sp. nov.	*lao*	*minhlei*	*gialaiensis* sp. nov.	*siamensis*	*somchanhae*	*melanostictus*
*Dixonius* sp.	**n/a**												
cf. siamensis	6.33	**n/a**											
*aaronbaueri*	18.52	18.37	**n/a**										
*taoi*	11.49	13.16	16.07	**0.01**									
*vietnamensis*	12.12	13.67	18.84	6.58.	**n/a**								
cf. vietnamensis	12.12	12.43	18.31	7.36	2.57	**n/a**							
*muangfuangensis* sp. nov.	10.78	8.17	18.17	11.36	12.79	12.50	**0.00**						
*lao*	8.46	9.26	16.66	10.90	11.90	11.39	3.10	**0.00**					
*minhlei*	13.97	15.33	17.56.	13.35	14.13	13.92	13.23	13.24	**n/a**				
*gialaiensis* sp. nov.	13.51	14.27	15.73	11.78	13.40	13.18	13.11	10.90	3.60	**0.00**			
*siamensis*	13.71	14.83	16.14	11.74	12.33	12.22	12.70	11.96	12.54	10.56	**0.00**		
*somchanhae*	13.31	12.66	17.73	12.90	12.57	12.40	12.40	12.27	12.24	10.63	9.07	**0.00**	
*melanostictus*	13.30	13.04	15.16	11.23	13.12	13.01	11.99	10.70	14.09	11.53	12.10	11.09	**n/a**

The time-calibrated BEAST analysis places the divergence between *Dixoniusaaronbaueri* and the remaining species of *Dixonius* at approximately 24.04 mya (20.23–27.68 highest posterior density [HPD]). Within the Vietnam’s lineages, *D.gialaiensis* sp. nov. and *D.minhlei* diverged from each other at approximately 3.19 mya (0.79–5.78 HPD) and within the Lao lineages, *D.muangfuangensis* sp. nov. and *D.lao* diverged approximately 3.47 mya (1.37–6.16 HPD) (Fig. 3).

### ﻿Statistical analyses

**Figure 4.**
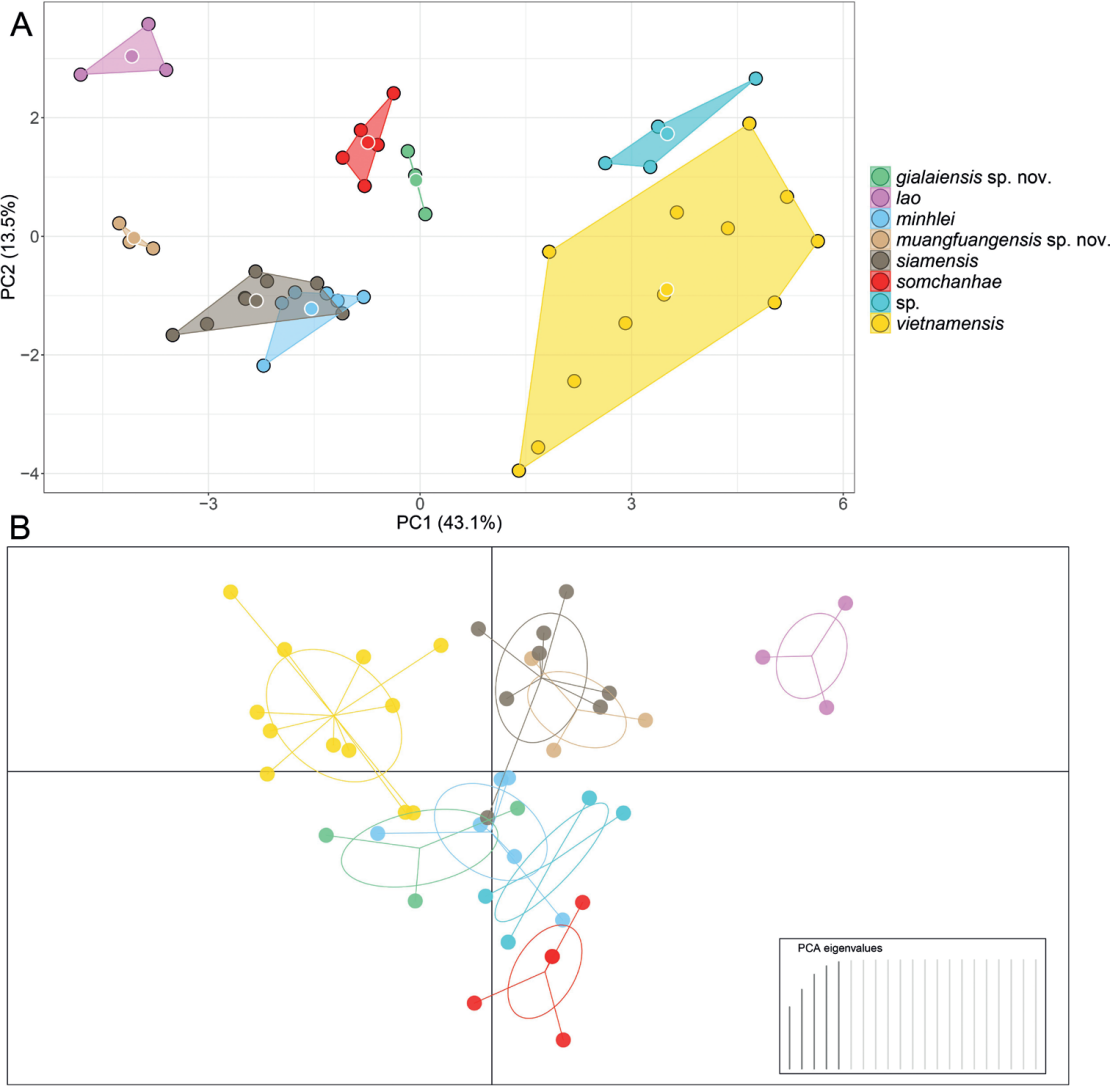
**A** principal component analysis (PCA) of *Dixonius* species showing their morphospatial relationships along the first two components based on normalized morphometric and meristic characters **B** discriminant analysis of principal components (DAPC) based on retention of the first five PCs with 67% inertia ellipsoids.

The first two principal components (PC1 and PC2) of the PCA analysis recovered 56.6% of the variation in the morphometric and meristic data set (Fig. 4A) and loaded most heavily for body width (BW), head width (HW), eye nostril distance (EN), eye snout distance (ES), and eye ear distance (EE) along PC1 and interorbital distance (IO), supralabials (SPLr/l), number of supralabial at midorbital position (MO), and ventral scales (V) along PC2 (Table 5). The PCA recovered *D.gialaiensis* sp. nov. and *D.muangfuangensis* sp. nov. to be widely separated from most other species with *D.muangfuangensis* sp. nov. only overlapping with the distantly related *D.siamensis*. The two distantly related new species are well-separated from most other species in the DAPC but each overlaps with one other species in their 67% inertia ellipses (Fig. 4B).

**Table 5.** Summary statistics of the principal component analysis of *Dixonius* species. Abbreviations are listed in the Materials and methods.

	**PC1**	**PC2**	**PC3**	**PC4**	**PC5**	**PC6**	**PC7**
**Standard deviation**	**3.01003227**	**1.685877698**	**1.23949927**	**1.189032683**	**1.136318219**	**0.950656207**	**0.922402779**
**Proportion of Variance**	**0.43144**	**0.13534**	**0.07316**	**0.06732**	**0.06149**	**0.04304**	**0.04052**
**Cumulative Proportion**	**0.43144**	**0.56678**	**0.63994**	**0.70727**	**0.76875**	**0.81179**	**0.85231**
**eigen**	9.060294267	2.842183612	1.53635844	1.413798721	1.291219096	0.903747223	0.850826887
**SVL**	-0.183137642	0.011423135	-0.069418522	0.076025214	-0.119546371	0.451176774	-0.589642945
**BW**	-0.287276767	0.064974951	0.187163981	-0.199453201	0.019777911	-0.133068566	-0.114357041
**HL**	-0.222534372	0.251387029	0.23514022	0.300194841	0.119329084	-0.056134295	0.150350725
**HW**	-0.264923454	0.100856053	0.274888978	0.257153297	-0.193697896	-0.025433828	0.20364848
**HD**	-0.239223187	-0.126312635	0.224210506	-0.024761051	-0.413575793	0.029259717	0.233903564
**EL**	-0.2480955	0.169750915	0.178082873	0.002208353	0.210266124	0.137233441	-0.232577889
**ED**	-0.202876478	0.122593123	0.079950567	-0.239727042	-0.47584928	0.235373894	-0.048044975
**EN**	-0.265593548	-0.130857091	-0.293077474	0.03772842	0.029146276	-0.105240363	0.142115916
**ES**	-0.267303156	-0.128737264	-0.102433066	-0.036514974	-0.068578553	-0.009743264	0.195931987
**EE**	-0.276238196	-0.150072094	-0.016576264	0.149081788	0.006956725	-0.271219422	-0.084638951
**IN**	-0.239210846	-0.181935095	0.070874242	0.114696597	0.170327297	0.022503069	0.138746934
**IO**	-0.131045671	-0.460675273	-0.164479294	-0.032535496	0.242758327	-0.169473493	-0.152061581
**FAr**	-0.279019143	-0.171574811	-0.122828868	-0.090763378	0.096076353	-0.023168457	0.04857391
**TBLr**	-0.256167278	-0.099347048	-0.096744886	-0.219547386	0.043230096	0.332696024	0.101539921
**AGr**	-0.262180808	-0.1304743	0.000207287	-0.261650023	-0.044763987	-0.216118201	-0.26353247
**SPLr.l**	-0.138456955	0.383331303	-0.225322477	0.206591458	0.113507526	0.110869199	-0.176892801
**IFLr.l**	-0.089464182	0.168661032	-0.585083828	0.180041929	-0.237863864	-0.199925005	-0.127174583
**MO**	-0.156905954	0.393579439	-0.305813247	0.010226561	-0.089299256	-0.164453012	0.254918394
**IOS**	-0.068091843	-0.230600144	0.078301763	0.673617186	0.047911701	0.164434462	-0.062199104
**V**	-0.140473075	0.310134826	0.27924011	-0.11493361	0.255279707	-0.405751544	-0.23957707
**T4r.l**	-0.152382721	0.157971329	-0.130436885	-0.183960387	0.490226326	0.396201891	0.316382072
	**PC8**	**PC9**	**PC10**	**PC11**	**PC12**	**PC13**	**PC14**
**Standard deviation**	**0.843138943**	**0.710443326**	**0.614017867**	**0.525772586**	**0.515133085**	**0.463343505**	**0.418149629**
**Proportion of Variance**	**0.03385**	**0.02403**	**0.01795**	**0.01316**	**0.01264**	**0.01022**	**0.00833**
**Cumulative Proportion**	**0.88616**	**0.91019**	**0.92815**	**0.94131**	**0.95395**	**0.96417**	**0.97249**
**eigen**	0.710883277	0.50472972	0.37701794	0.276436812	0.265362095	0.214687204	0.174849113
**SVL**	0.265288193	-0.446853384	0.196416397	-0.110376596	0.139472173	-0.13673416	0.085656549
**BW**	0.09057594	-0.129906618	-0.173199899	0.177854897	0.108703018	-0.085524495	-0.103253386
**HL**	-0.070645969	-0.213980283	-0.087846741	-0.389903504	-0.321747003	-0.17842853	-0.021701889
**HW**	0.039378469	-0.022086396	0.070374143	0.022285485	0.134276418	-0.209651757	0.058151643
**HD**	0.139854316	-0.180680891	-0.127550531	0.157737189	-0.027411952	0.137530567	-0.134451774
**EL**	-0.203202698	0.278604397	-0.198153958	0.402630535	-0.109054652	-0.264792874	0.164044114
**ED**	-0.319474696	0.251272882	-0.008164379	0.059050615	-0.034208937	0.225488518	0.158104491
**EN**	0.295981088	0.09576584	0.048312128	0.019723407	0.172978909	0.06220716	-0.41905017
**ES**	0.426919556	0.259878689	-0.007215665	-0.131144367	0.25121175	-0.129629771	0.403591724
**EE**	0.038959782	-0.113710926	-0.227904855	0.339050813	-0.133303008	-0.064748891	-0.201696035
**IN**	-0.469863359	-0.267676841	-0.188020288	-0.317994993	0.412756086	0.253021631	0.111955286
**IO**	-0.122814373	0.056652546	-0.125361847	0.03792394	0.095742055	-0.182302581	0.359575483
**FAr**	-0.057154891	0.014271255	0.303625758	-0.210415667	-0.567849769	-0.191700829	-0.00332367
**TBLr**	-0.266800377	0.279797707	0.228807449	-0.14978157	0.145371473	-0.238209022	-0.413156342
**AGr**	0.069118103	0.008227511	-0.004079106	-0.18336757	-0.303043068	0.52714368	0.090386865
**SPLr.l**	0.178898959	0.359957261	-0.462995625	-0.293841774	0.004054536	0.173258047	-0.103413131
**IFLr.l**	-0.352501894	-0.193922056	0.002786908	0.157568267	0.04772871	-0.046397338	-0.113553524
**MO**	-0.000619521	-0.057816313	0.271775541	0.090085724	0.002030737	-0.003433238	0.40989474
**IOS**	-0.02790961	0.257813239	0.336314946	0.217437021	-0.064780745	0.352986111	0.03220845
**V**	-0.010841335	0.086524979	0.460243812	-0.002655612	0.309267431	0.129809552	-0.119165889
**T4r.l**	0.091314647	-0.276112686	0.005762572	0.333431445	-0.07395688	0.301989669	0.038025431
	**PC15**	**PC16**	**PC17**	**PC18**	**PC19**	**PC20**	**PC21**
**Standard deviation**	**0.376199721**	**0.365477475**	**0.339179752**	**0.282916626**	**0.236187037**	**0.171149685**	**0.149480188**
**Proportion of Variance**	**0.00674**	**0.00636**	**0.00548**	**0.00381**	**0.00266**	**0.00139**	**0.00106**
**Cumulative Proportion**	**0.97923**	**0.98559**	**0.99107**	**0.99488**	**0.99754**	**0.99894**	**1**
**eigen**	0.14152623	0.133573785	0.115042904	0.080041817	0.055784316	0.029292215	0.022344327
**SVL**	-0.083499418	0.052323475	-0.106015238	0.003344968	-0.031996278	0.03916795	-0.019457555
**BW**	-0.178247777	-0.058497317	0.597846907	-0.255954135	0.379366422	-0.199217481	0.221418054
**HL**	0.08265144	-0.13083192	-0.024132742	0.014837636	0.131660321	0.479388229	0.2891919
**HW**	0.244724903	-0.059011694	0.113956101	-0.074931386	0.04577337	-0.069612504	-0.732492773
**HD**	0.050630806	0.316234183	0.16660119	0.299515683	-0.49781424	0.046244883	0.219619924
**EL**	0.296740909	0.376559691	-0.260809485	-0.089676204	-0.027290387	-0.071210438	0.140852117
**ED**	-0.231235089	-0.168155577	-0.115371244	0.284151752	0.361474786	0.202917841	-0.049017145
**EN**	0.022603597	0.420837275	-0.236893263	0.02900181	0.436157574	0.238920063	-0.045962367
**ES**	0.248024801	-0.355459446	-0.140327076	0.068202164	0.014098586	-0.168841736	0.325882917
**EE**	-0.393037351	-0.425564191	-0.418495874	-0.066368342	-0.173649977	-0.04666024	-0.080329
**IN**	-0.081626216	0.152259274	-0.192312604	-0.041856001	0.036286384	-0.306664296	0.054524547
**IO**	-0.115305892	0.069986307	0.310505175	0.23842857	-0.096262685	0.459593261	-0.180632462
**FAr**	-0.116757716	0.126720851	0.055407627	0.30600813	0.081267972	-0.470097472	-0.064137815
**TBLr**	-0.021197578	-0.178071358	0.100589652	-0.310374261	-0.331807871	0.142300685	0.047913831
**AGr**	0.334353255	-0.016725971	-0.026314358	-0.397995653	-0.105887039	0.100382932	-0.137085205
**SPLr.l**	-0.184269598	0.036899566	0.194841372	0.163667167	-0.204455175	-0.121267207	-0.156153276
**IFLr.l**	0.428551026	-0.165166993	0.155004291	0.142458525	0.008969802	-0.065554124	0.111490515
**MO**	-0.391157106	0.255079755	-0.004834981	-0.342258191	-0.175496507	0.081476947	0.028734251
**IOS**	-0.059743664	-0.04851249	0.20733712	-0.095515858	0.057436279	-0.015186946	0.173384569
**V**	0.002458015	-0.062420243	-0.039680685	0.361422477	-0.145522506	0.02608135	0.040083497
**T4r.l**	0.117972701	-0.206198025	0.0622901	0.17118706	0.012651195	0.069479213	-0.122746338

**Figure 5.**
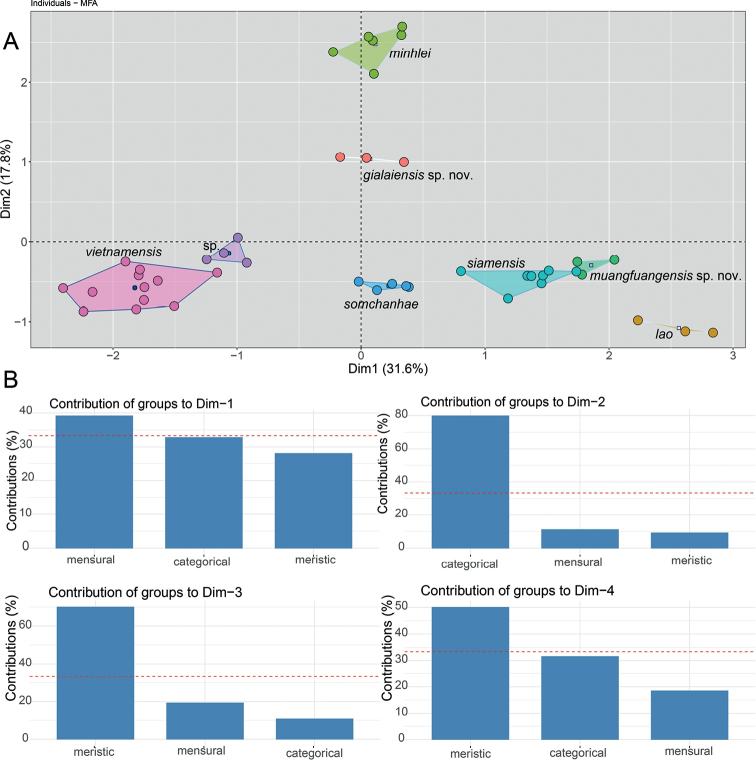
**A**MFA scatter plot showing the morphospatial relationships among the *Dixonius* species based on normalized morphometric, meristic, and color pattern characters **B** bar graphs showing the percent contribution of each data type to the overall variation in the data dimensions 1–4. The dashed red line in the bar graphs indicates the expected average value if the contributions of each data type were equal.

The MFA analysis recovered all species to be separated from one another including *Dixoniusmuangfuangensis* sp. nov. and *D.siamensis* (Fig. 5A). The morphometric data contributed to approximately 40% of the variation along Dim-1 followed by the categorical and meristic data. For Dim-2, the categorical data contributed 80% of the variation followed by morphometric and meristic data. Dim-3 showed that meristic data contributed 70% of the variation followed by morphometric and categorical data (Fig. 5B).

**Figure 6.**
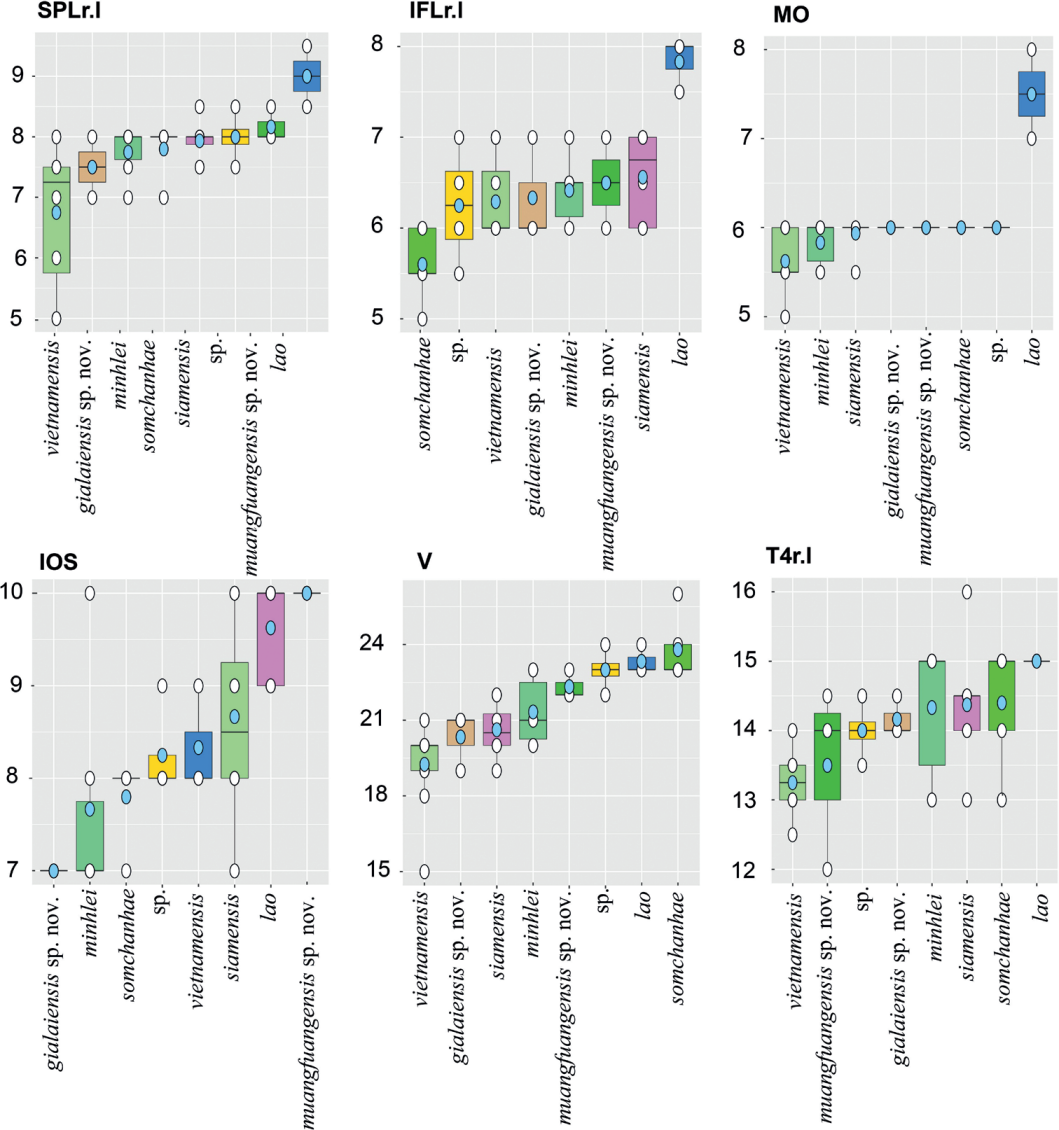
Boxplot comparisons of meristic characters among the *Dixonius* species where interspecific statistical differences were recovered (see Table 7). Pale blue circles are means and the black horizontal bars are medians.

**Figure 7.**
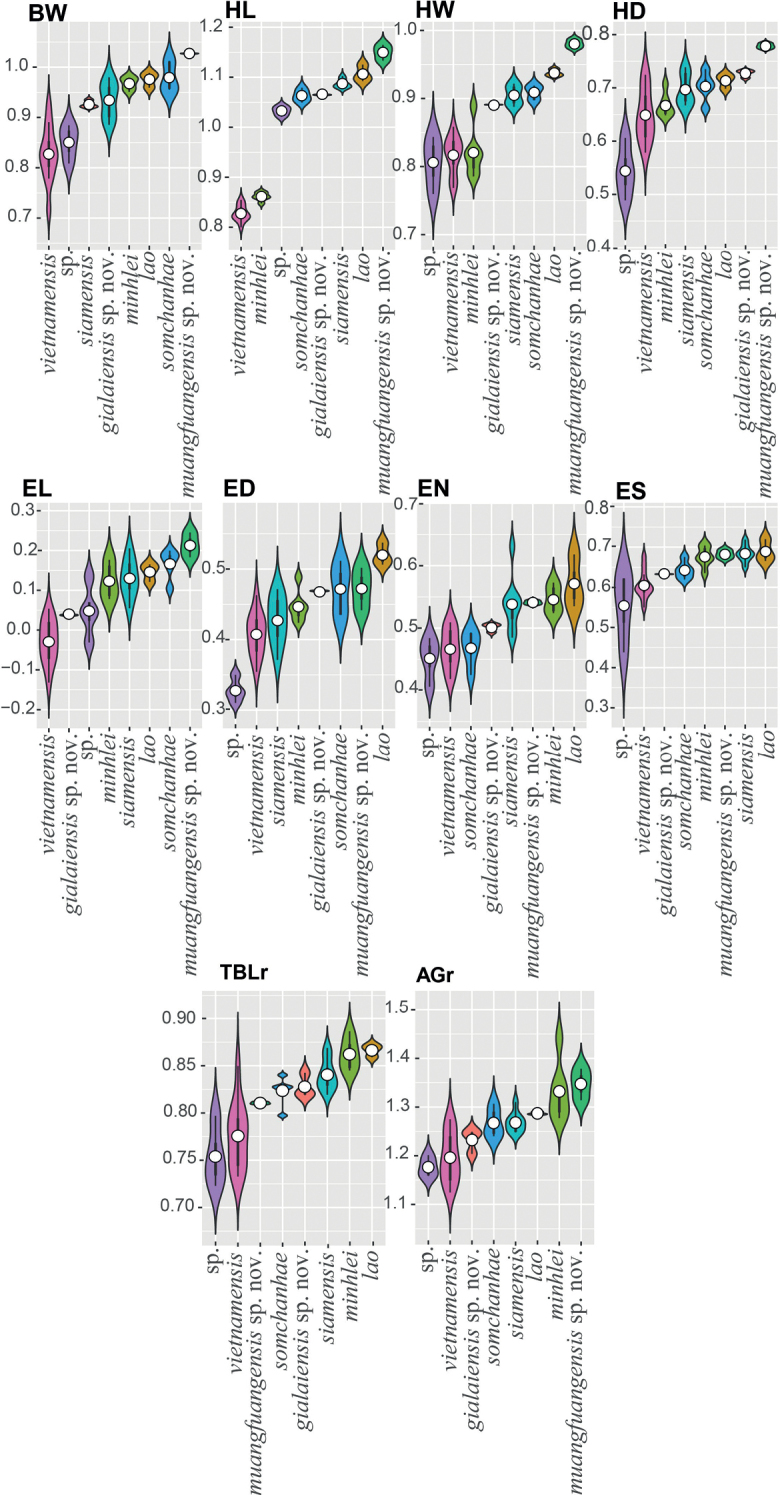
Violin plots of the normalized morphometric characters overlain with box plots showing the range, frequency, mean (white dot), and 50% quartile (black rectangle) of characters where interspecific statistical differences were recovered (see Table 7). New species in bold italics.

The ANOVAs and subsequent TukeyHDS tests demonstrated that *Dixoniusgialaiensis* sp. nov. bears statistically different mean values between it and all other species in various combinations of characters (Tables 6, 7) and differs significantly from its sister species *D.minhlei* in head length (HL mean = 1.07 vs. 0.86, *p* = 0.000, respectively), in head width (HW mean = 0.89 vs. 0.82, *p* = 0.005, respectively), and in axilla to groin length, (AGr mean = 1.23 vs 1.32 *p* = 0.022) (Fig. 5; Tables 6, 7). *Dixoniusmuangfuangensis* sp. nov. also differed significantly from all other species in various combinations of characters and from its sister species *D.lao* it differs in head length (HL mean = 1.15 vs. 1.11, *p* = 0.004, respectively) and numbers of infralabials (IFL mean = 6.50 vs. 7.83, *p* = 0.026, respectively), and in numbers of supralabial at midorbital position (MO mean = 6.00 vs. 7.50, *p* = 0.00001, respectively) (Fig. 5, Tables 6, 7). Variation in all metric characters are visualized in Figs 6, 7.

**Table 6.** Summary statistics of morphometric and meristic characters among the *Dixonius* species.

Species	SVL	BW	HL	HW	HD	EL	ED	EN	ES	EE	IN	IO	FAr	TBLr	AGr	SPLr.l	IFLr.l	MO	IOS	V	T4r.l
***Dixoniusgialaiensis* sp. nov. (*n* = 3)**																					
Mean	1.62	0.93	1.07	0.89	0.73	0.04	0.47	0.50	0.63	0.51	0.14	0.11	0.75	0.83	1.23	7.5	6.33	6	7	20.33	14.17
SD	0.060	0.007	0.003	0.001	0.007	0.005	0.002	0.007	0.002	0.005	0.018	0.029	0.034	0.013	0.025	0.5	0.577	0	0	1.155	0.289
Lower	1.56	0.92	1.06	0.89	0.72	0.04	0.47	0.49	0.63	0.51	0.12	0.08	0.73	0.82	1.20	7	6	6	7	19	14
Upper	1.68	0.93	1.07	0.89	0.73	0.05	0.47	0.50	0.64	0.52	0.15	0.13	0.79	0.84	1.25	8	7	6	7	21	14.5
***D.lao* (*n* = 3)**																					
Mean	1.67	0.98	1.11	0.94	0.71	0.15	0.52	0.57	0.69	0.57	0.18	0.16	0.79	0.87	1.29	9	7.83	7.5	8.33	23.33	15
SD	0.099	0.014	0.017	0.005	0.013	0.022	0.016	0.042	0.028	0.012	0.013	0.047	0.017	0.007	0.003	0.5	0.289	0.5	0.577	0.577	0
Lower	1.55	0.96	1.09	0.93	0.70	0.12	0.51	0.54	0.67	0.56	0.17	0.12	0.78	0.86	1.29	8.5	7.5	7	8	23	15
Upper	1.74	0.99	1.13	0.94	0.72	0.17	0.54	0.62	0.72	0.58	0.20	0.21	0.81	0.87	1.29	9.5	8	8	9	24	15
***D.minhlei* (*n* = 6)**																					
Mean	1.65	0.97	0.86	0.82	0.67	0.12	0.45	0.55	0.68	0.56	0.17	0.57	0.80	0.86	1.33	7.75	6.42	5.83	7.67	21.33	14.33
SD	0.025	0.012	0.008	0.037	0.022	0.044	0.022	0.021	0.025	0.022	0.023	0.025	0.020	0.017	0.060	0.418	0.376	0.258	1.211	1.366	1.033
Lower	1.61	0.95	0.85	0.79	0.65	0.08	0.42	0.52	0.64	0.53	0.14	0.53	0.78	0.85	1.28	7	6	5.5	7	20	13
Upper	1.68	0.98	0.87	0.89	0.71	0.18	0.49	0.57	0.70	0.59	0.21	0.60	0.83	0.89	1.44	8	7	6	10	23	15
***Dixoniusmuangfuangensis* sp. nov. (*n* = 3)**																					
Mean	1.69	1.03	1.15	0.99	0.78	0.21	0.47	0.54	0.68	0.66	0.19	0.33	0.78	0.81	1.35	8.17	6.5	6	10	22.33	13.5
SD	0.096	0.001	0.016	0.006	0.005	0.031	0.030	0.003	0.011	0.005	0.011	0.006	0.004	0.001	0.032	0.289	0.5	0	0	0.577	1.323
Lower	1.58	1.03	1.13	0.97	0.77	0.18	0.44	0.54	0.67	0.65	0.18	0.32	0.78	0.81	1.31	8	6	6	10	22	12
Upper	1.75	1.03	1.16	0.99	0.78	0.25	0.50	0.54	0.69	0.66	0.20	0.34	0.78	0.81	1.38	8.5	7	6	10	23	14.5
***D.siamensis* (*n* = 8)**																					
Mean	1.62	0.93	1.09	0.90	0.70	0.13	0.43	0.54	0.68	0.58	0.25	0.54	0.80	0.84	1.27	7.94	6.56	5.94	9.63	20.63	14.38
SD	0.077	0.0357	0.009	0.014	0.029	0.048	0.034	0.044	0.021	0.031	0.046	0.016	0.028	0.017	0.021	0.320	0.496	0.177	0.518	1.061	0.835
Lower	1.61	0.95	0.85	0.79	0.65	0.08	0.42	0.52	0.64	0.53	0.14	0.53	0.78	0.85	1.23	7	6	5.5	7	20	13
Upper	1.68	0.987	0.87	0.89	0.71	0.18	0.49	0.57	0.70	0.59	0.21	0.60	0.83	0.89	1.44	8	7	6	10	23	15
***D.somchanhae* (*n* = 6)**																					
Mean	1.62	0.97	1.07	0.91	0.70	0.17	0.46	0.48	0.65	0.50	0.18	0.23	0.73	0.83	1.26	7.75	5.67	6	8.17	23.33	14.67
SD	0.045	0.033	0.013	0.009	0.026	0.034	0.043	0.041	0.024	0.023	0.073	0.150	0.050	0.016	0.027	0.418	0.408	0	0.983	1.633	1.033
Lower	1.55	0.93	1.05	0.90	0.66	0.10	0.40	0.42	0.62	0.47	0.098	0.13	0.68	0.80	1.24	7	5	6	7	21	13
Upper	1.67	1.01	1.08	0.92	0.74	0.20	0.51	0.55	0.68	0.54	0.29	0.53	0.82	0.84	1.31	8	6	6	10	26	16
***D.* sp. (*n* = 4)**																					
Mean	1.55	0.85	1.03	0.81	0.54	0.05	0.33	0.45	0.55	0.49	0.11	0.21	0.66	0.75	1.18	8	6.25	6	8.25	23	14
SD	0.083	0.033	0.009	0.037	0.049	0.068	0.017	0.033	0.087	0.033	0.007	0.015	0.032	0.032	0.020	0.408	0.645	0	0.5	0.816	0.408
Lower	1.48	0.81	1.02	0.76	0.49	-0.03	0.31	0.41	0.44	0.44	0.11	0.19	0.62	0.72	1.16	7.5	5.5	6	8	22	13.5
Upper	1.66	0.88	1.04	0.84	0.61	0.13	0.35	0.48	0.62	0.52	0.12	0.22	0.70	0.80	1.20	8.5	7	6	9	24	14.5
***D.vietnamensis* (*n* = 12)**																					
Mean	1.56	0.83	0.83	0.82	0.65	-0.03	0.41	0.47	0.61	0.48	0.10	0.29	0.67	0.78	1.20	6.75	6.29	5.63	8.67	19.25	13.25
SD	0.088	0.055	0.016	0.028	0.049	0.054	0.030	0.031	0.035	0.055	0.070	0.165	0.063	0.035	0.051	1.177	0.450	0.377	0.985	1.545	0.399
Lower	1.41	0.72	0.80	0.77	0.58	-0.13	0.35	0.42	0.55	0.36	0.02	0.12	0.55	0.73	1.12	5	6	5	7	15	12.5
Upper	1.66	0.92	0.85	0.86	0.72	0.05	0.46	0.51	0.68	0.55	0.28	0.53	0.76	0.85	1.27	8	7	6	10	21	14

**Table 7.** Significant *p*-values from the results of the ANOVA and TukeyHDS analyses comparing all combinations of species pairs. Character abbreviations are listed in the Materials and methods.

**Morphometric characters**	**BW**	**HL**	**HW**	**HD**	**EL**	**ED**	**EN**	**ES**	**FAr**	**TBLr**	**AGr**
*lao* vs. *gialaiensis* sp. nov.		0.007									
*minhlei* vs. *gialaiensis* sp. nov.		0.00	0.005								0.022
*muangfuangensis* sp. nov. vs. *gialaiensis* sp. nov.	0.040	< 0.001	0.001		0.001						0.023
*siamensis* vs. *gialaiensis* sp. nov.											
*somchanhae* vs. *gialaiensis* sp. nov.					0.016						
sp. vs. *gialaiensis* sp. nov.		0.021	0.001	< 0.001		< 0.001				0.006	
*vietnamensis* vs. *gialaiensis* sp. nov.	0.005	0.00	< 0.001	0.030		0.040				0.036	
*minhlei* vs. *lao*		0.00	< 0.001			0.017					
*muangfuangensis* sp. nov. vs. *lao*		0.003									
*siamensis* vs. *lao*						< 0.001					
*somchanhae* vs. *lao*		< 0.001					0.002				
sp. vs. lao	0.002	< 0.001	< 0.001	< 0.001		< 0.001	< 0.001	< 0.001	0.005	< 0.001	0.018
*vietnamensis* vs. *lao*	< 0.001	0.00	< 0.001		< 0.001	< 0.001	< 0.001	0.017	0.001	< 0.001	0.023
*muangfuangensis* sp. nov. vs. *minhlei*		0.00	< 0.001	0.002							
*siamensis* vs. *minhlei*		0.00	< 0.001								
*somchanhae* vs. *minhlei*		0.00	< 0.001				0.006		0.035		
sp. vs. *minhlei*	< 0.001	0.00		< 0.001		< 0.001	0.001	< 0.001	< 0.001	< 0.001	< 0.001
*vietnamensis* vs. *minhlei*	< 0.001	< 0.001			< 0.001		< 0.001	0.007	< 0.001	< 0.001	< 0.001
*siamensis* vs. *muangfuangensis* sp. nov.	0.016	< 0.001	0.001	0.030							
*somchanhae* vs. *muangfuangensis* sp. nov.		<0.001	0.006								
sp. vs. *muangfuangensis* sp. nov.	< 0.001	< 0.001	< 0.001	< 0.001	0.001	< 0.001	0.016	0.001	0.013		< 0.001
*vietnamensis* vs. *muangfuangensis* sp. nov.	<0.001	0.00	<0.001	<0.001	<0.001	0.021	0.019	0.038	0.004		<0.001
*somchanhae* vs. *siamensis*		0.031					0.010		0.018		
sp. vs. *siamensis*	0.016	< 0.001	< 0.001	< 0.001		< 0.001	0.002	< 0.001	< 0.001	< 0.001	0.012
*vietnamensis* vs. *siamensis*	< 0.001	0.00	< 0.001		< 0.001		< 0.001	< 0.001	< 0.001	< 0.001	0.007
sp. vs. *somchanhae*	< 0.001	0.017	< 0.001	< 0.001	0.013	< 0.001		0.017		0.003	0.032
*vietnamensis* vs. *somchanhae*	< 0.001	0.00	< 0.001		< 0.001	0.003				0.013	0.038
*vietnamensis* vs. sp.		0.00		< 0.001		< 0.001					
**Morphometric characters**	**SPLr.l**	**IFLr.l**	**MO**	**IOS**	**V**	**T4r.l**					
*lao* vs. *gialaiensis* sp. nov.		0.008	< 0.001								
*minhlei* vs. *gialaiensis* sp. nov.											
*muangfuangensis* sp. nov. vs. *gialaiensis* sp. nov.				< 0.001							
*siamensis* vs. *gialaiensis* sp. nov.				< 0.001							
*somchanhae* vs. *gialaiensis* sp. nov.					0.011						
sp. vs. *gialaiensis* sp. nov.											
*vietnamensis* vs. *gialaiensis* sp. nov.				0.041							
*minhlei* vs. *lao*		0.003	< 0.001								
*muangfuangensis* sp. nov. vs. *lao*		0.026	< 0.001								
*siamensis* vs. *lao*		0.007	< 0.001								
*somchanhae* vs. *lao*		< 0.001	< 0.001								
sp. vs. *lao*		0.002	< 0.001								
*vietnamensis* vs. *lao*	< 0.001	< 0.001	< 0.001		< 0.001	0.015					
*muangfuangensis* sp. nov. vs. *minhlei*				0.004							
*siamensis* vs. *minhlei*				0.001							
*somchanhae* vs. *minhlei*					0.045						
sp. vs. *minhlei*											
*vietnamensis* vs. *minhlei*					0.038						
*siamensis* vs. *muangfuangensis* sp. nov.											
**Morphometric characters**	**SPLr.l**	**IFLr.l**	**MO**	**IOS**	**V**	**T4r.l**					
*somchanhae* vs. *muangfuangensis* sp. nov.				0.010							
sp. vs. *muangfuangensis* sp. nov.											
*vietnamensis* vs. *muangfuangensis* sp. nov.					0.011						
*somchanhae* vs. *siamensis*		0.019			0.002						
sp. vs. *siamensis*					0.065						
*vietnamensis* vs. *siamensis*	0.022					0.035					
sp. vs. *somchanhae*											
*vietnamensis* vs. *somchanhae*					< 0.001						
*vietnamensis* vs. sp.					< 0.001						

## ﻿Taxonomy

### ﻿ Dixonius gialaiensis sp. nov.

Taxon classificationAnimaliaSquamataGekkonidae

63620ADC-A9D5-5D0D-8FE4-143C79AC4A51

https://zoobank.org/10BF67E1-8059-47CE-891C-B219BD7AA9C1

**Figure 8.**
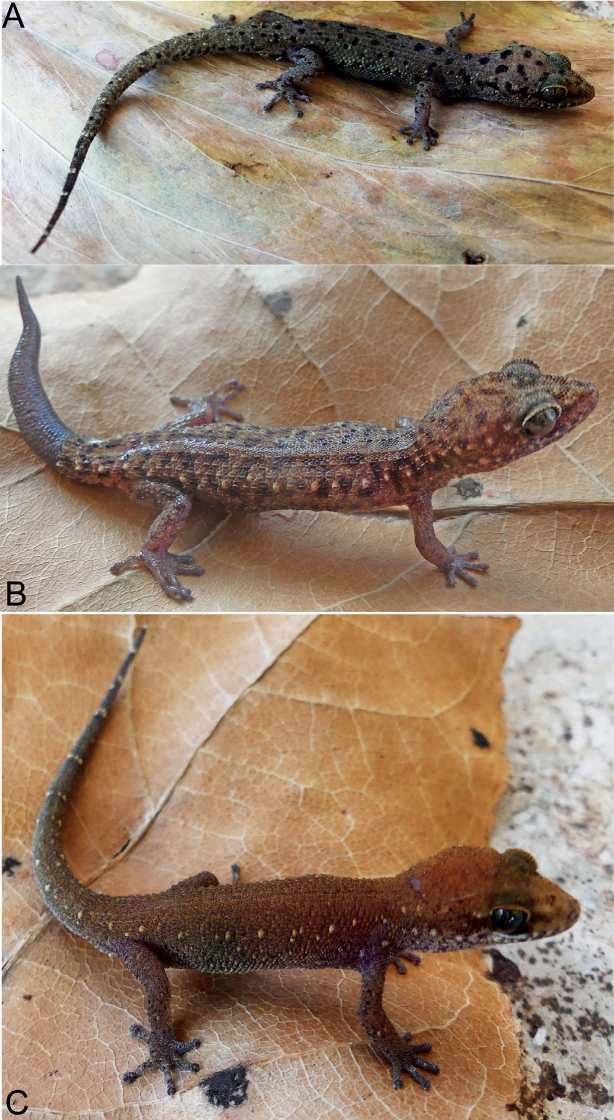
Dorsal views of *Dixoniusgialaiensis* sp. nov. **A** adult male holotype VNUF R.2020.22 (Field no. GL02) **B** adult female paratype VNUF R.2020.33 (Field No. GL03) **C** juvenile male paratype VNUF R.2020.44 (Field No. GL04) in Chu Se Mountain Pass, Hbong Commune, Chu Se District, Gia Lai Province.

Fig. 8 Gialai leaf-toed gecko

#### Material examined.

***Holotype*.** Adult male, VNUF R.2020.22 (Field no. GL02) in Chu Se Mountain Pass, H’Bong Commune, Chu Se District, Gia Lai Province (13°34'44.3"N, 108°13'55.7"E; 330 m a.s.l.), collected by Oanh Van Lo and Khanh Quoc Nguyen on 15 February 2020. ***Paratypes*.**VNUF R.2020.44 (Field No. GL04), juvenile male, and VNUF R.2020.33 (Field No. GL03), adult female; the same data as the holotype.

#### Diagnosis.

*Dixoniusgialaiensis* sp. nov. can be separated from all other species of *Dixonius* by possessing the unique combination of having a maximum SVL of 47.4 mm; 19 longitudinal rows of dorsal tubercles at midbody; 19–21 longitudinal rows of ventrals across the abdomen; 7 or 8 supralabials, sixth in at midorbital position; 6 or 7 infralabials; 7 interorbital scales; 7 or 8 precloacal pores in males, femoral pores lacking; precloacal and femoral pores absent in female; 13–15 lamellae on fourth toe; dorsum olive grey color with more round brown blotches; canthal stripe continues behind orbit to back of head; lips with dark bars; two regularly disposed whitish tubercles along the sides near the flanks to tail tip. These characters are scored across all *Dixonius* species from Vietnam and Laos in Tables 6, 7.

#### Description of the holotype.

Adult male, SVL 41.2 mm; head moderate in length (HL/SVL 0.28), wide (HW/HL 0.66), depressed (HD/HL 0.44), distinct from neck; prefrontal region concave; canthus rostralis rounded; snout elongate (ES/HL 0.37), rounded in dorsal profile; eye moderate size (ED/HL 0.25); ear opening oval, obliquely oriented, moderate in size; diameter of eye slightly smaller than eye to ear distance (ED/EE 0.88); rostral rectangular, partially divided dorsally by straight rostral groove, bordered posteriorly by large left and right supranasals, bordered laterally by first supralabials; external nares bordered anteriorly by rostral, dorsally by large supranasal, posteriorly by two smaller postnasals, bordered ventrally by first supralabial; 8,7 (R,L) rectangular supralabials extending to below and slightly past posterior margin of eye, sixth in midorbital position; 6,6 (R,L), infralabials tapering smoothly to just below midpoint of eye, decreasing gradually in size; scales of rostrum and lores flat to domed, larger than granular scales on top of head and occiput; scales of occiput intermixed with distinct, small, conical tubercles; superciliaries elongate, largest anteriorly; mental triangular, bordered laterally by first infralabials and posteriorly by large left and right trapezoidal postmentals contacting medially for 60% of their length posterior to mental; gular and throat scales small, granular, grading anteriorly into slightly larger, flatter, smooth, imbricate, pectoral and ventral scales.

Body relatively short (AG/SVL 0.38); dorsal scales small, granular interspersed with larger, conical, regularly arranged, keeled tubercles; tubercles extend from top of head onto posterior haft of tail forming longitudinal rows, terminating at last portion of tail; smaller tubercles extend anteriorly onto nape and occiput, diminishing in size and distinction on top of head; 19 longitudinal rows of tubercles at midbody; 33 paravertebral scales, number of scales in a paravertebral row from first scale posterior to parietal scale to last scale at the level of vent opening; 23 paravertebral scales in a row between limb insertions; 21 flat, imbricate, ventral scales much larger than dorsal scales; 7 enlarge, pore-bearing, precloacal scales in an angular series; and no deep precloacal groove or depression.

Forelimbs moderate in stature, relatively short (FA/SVL 0.15); granular scales of forearm slightly larger than those on body, interspersed with small tubercles; hind limbs more robust than forelimbs, moderate in length (TBL/SVL 0.17), covered dorsally by granular scales interspersed with large, and small conical tubercles; ventral scales of thigh flat, imbricate, larger than dorsals; subtibial scales flat, imbricate; proximal femoral scales smaller than distal femorals; femoral pores absent; digits relatively long with 14 lamellae on fourth toe; and claws well developed.

Tail 108.4 mm in length, 4.5 mm in width at base, tapering to a point; dorsal scales of flat, square with conical, keeled tubercles in anterior part; median row of transversely expanded subcaudal scales, significantly larger than dorsal caudal scales on original portion; base of tail bearing hemipenal swellings; and postcloacal scales flat, imbricate.

#### Coloration in life

**(Fig. 8).** Ground color of dorsal head and dorsum grey brown with rounded black-brown blotches, decreasing gradually in size from head to body; canthal stripe continues behind orbit to back of head; dark bars on the lips; uneven light spots running from postorbital along the flanks to tip tail; upper surface of fore- and hindlimbs uniformly light grey with black-brown spots; dorsum of tail covered with some small rounded black-brown blotches; ventral surface beige uniformly as the belly and the throat.

#### Variation

**(Fig. 8).** The female paratype (VNUF R.2020.33) generally has more dark brown blotches on head and dorsum, and uniformly black on the new regenerated tail. The dorsum of the of head and body of the juvenile male paratype (VNUF R.2020.44) pale brown with pale-colored blotches on granulose skin arranged along its sides extending from the flanks to the tail tip. Further measurements are summarized in Tables 2–4, Suppl. material 1: table S1.

#### Distribution.

*Dixoniusgialaiensis* sp. nov. currently is only known from the type locality of Chu Se Mountain Pass, H’Bong Commune, Chu Se District, Gia Lai Province, Central Highlands, Vietnam (Fig. 1).

#### Natural history.

**Figure 9.**
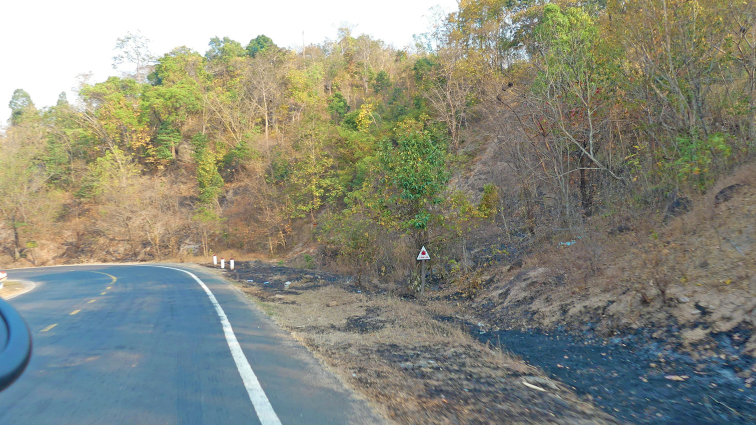
Habitat of *Dixoniusgialaiensis* sp. nov. HBong Commune, Chu Se District, Gia Lai Province, Central Highlands, Vietnam.

The specimens were found at night, between 19:45 and 21:00 h, on the ground in an area along the National Highway 25. The surrounding habitat was secondary montane forest with woody trees. The temperature and humidity were approximately 32.6 °C and 57% (Fig. 9).

#### Etymology.

The new species is named after the type locality of Gia Lai Province, Central Highlands, Vietnam.

#### Comparisons.

*Dixoniusgialaiensis* sp. nov. is the sister species to *D.minhlei* (Fig. 2) from which it differs by an uncorrected pairwise sequence divergence of 3.60% (Table 4). It is differentiated from it morphologically by having a significantly higher mean number of head length (HL), head width (HW), and axilla to groin length (AG). In addition, it differs from *D.minhlei* in color pattern (grey-brown dorsum with more round black-brown blotches versus olive gray dorsum with round brownish olive blotches). Statistically significant and discrete categorical differences between *Dixoniusgialaiensis* sp. nov. and all other species and populations are presented in Tables 5–7.

### ﻿ Dixonius muangfuangensis sp. nov.

Taxon classificationAnimaliaSquamataGekkonidae

155D254B-AAF9-5D44-B698-F6591AB96E89

https://zoobank.org/A447EC01-F653-4FE5-A616-5FBD25F027C6

**Figure 10.**
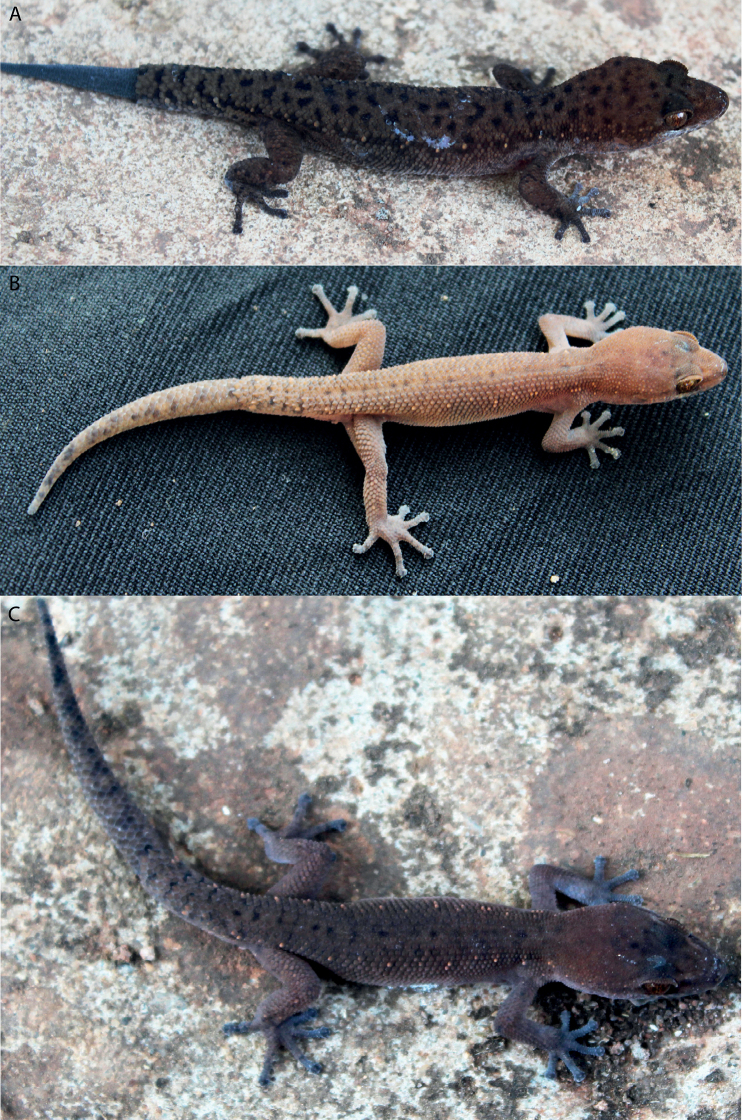
View of *Dixoniusmuangfuangensis* sp. nov. **A** adult male holotype VNUF R.2020.42 (Field no. MF.02) **B** adult female paratype VNUF R.2020.52 (Field no. MF. 03) **C** juvenile male paratype NUOL R.2022.01 (Field no. MF. 01) in Nadan Village, Muangfuang District, Vientiane Province, Central Laos.

Fig. 10 Muangfuang leaf-toed gecko

#### Material examined.

***Holotype*.** Adult male, VNUF R.2020.42 (Field no. MF.02) in Sinxay Temple, Nadan Village, Muangfuang District, Vientiane Province, Central Laos (18°32'52"N, 101°58'31"E; 276 m a.s.l.), collected by Saly Sitthivong and Thuong Huyen Nguyen on 05 December 2020. ***Paratypes*.**NUOL R.2022.01 (Field no. MF. 01), juvenile male, and VNUF R.2020.52 (Field no. MF. 03), adult female; the same data as given for the holotype.

#### Diagnosis.

*Dixoniusmuangfuangensis* sp. nov. can be separated from all other species of *Dixonius* by possessing the unique combination of having a maximum SVL of 56.7 mm; 21–23 longitudinal rows of dorsal tubercles at midbody; 20 or 21 longitudinal rows of ventrals across the abdomen; 7 or 8 supralabials, sixth in at midorbital position; 6 or 7 infralabials; 7 interorbital scales; 7 or 8 precloacal pores in males, femoral pores lacking; precloacal and femoral pores absent in female; 15 lamellae on fourth toe; dorsum olive grey color with numerous small and irregular black blotches; head with brown spots; light spots irregularly arranged from the back of the head to base of tail; lips with dark bars; two regularly disposed whitish tubercles on each side on each side. These characters are scored across all *Dixonius* species from Vietnam and Laos in Tables 6, 7.

#### Description of the holotype.

Adult male, SVL 55.6 mm; head moderate in length (HL/SVL 0.28), wide (HW/HL 0.71), depressed (HD/HL 0.45), distinct from neck; prefrontal region concave; canthus rostralis rounded; snout elongate (ES/HL 0.39), rounded in dorsal profile; eye moderate size (ED/HL 0.20); ear opening oval, obliquely oriented, moderate in size; diameter of eye much smaller than eye to ear distance (ED/EE 0.59); rostral rectangular, partially divided dorsally by straight rostral groove, bordered posteriorly by large left and right supranasals, bordered laterally by first supralabials; external nares bordered anteriorly by rostral, dorsally by large supranasal, posteriorly by two smaller postnasals, bordered ventrally by first supralabial; 8,8 (R,L) rectangular supralabials extending to below midpoint of eye, sixth in midorbital position; 7,7 (R,L), infralabials tapering smoothly to be just slightly past posterior below midpoint of eye, decreasing gradually in size; scales of rostrum and lores flat to domed, larger than granular scales on top of head and occiput; scales of occiput intermixed with distinct, small, conical tubercles; superciliaries elongate, largest anteriorly; mental triangular, bordered laterally by first infralabials and posteriorly by large left and right parallelogram postmentals contacting medially for 60% of their length posterior to mental; gular and throat scales small, granular, grading anteriorly into slightly smaller, flatter, smooth, imbricate, pectoral and ventral scales.

Body relatively short (AG/SVL 0.42) with well-defined ventrolateral folds; dorsal scales small, granular interspersed with moderate, conical, regularly arranged, keeled tubercles; tubercles extend from top of head onto interior haft of tail forming longitudinal rows, terminating at regenerated portion of tail; smaller tubercles extend anteriorly onto nape and occiput, diminishing in size and distinction on top of head; 23 longitudinal rows of tubercles at midbody; 45 paravertebral scales, number of scales in a paravertebral row from first scale posterior to parietal scale to last scale at the level of vent opening; 24 paravertebral scales in a row between limb insertions; 20 flat, imbricate, ventral scales much larger than dorsal scales; 8 enlarge, pore-bearing, precloacal scales in an angular series; and no deep precloacal groove or depression.

Forelimbs moderate in stature, relatively short (FA/SVL 0.12); granular scales of forearm slightly larger than those on body, interspersed with small tubercles; hind limbs more robust than forelimbs, moderate in length (TBL/SVL 0.13), covered dorsally by granular scales interspersed with large, and small conical tubercles; ventral scales of thigh flat, imbricate, larger than dorsals; subtibial scales flat, imbricate; proximal femoral scales smaller than distal femorals; femoral pores absent; digits relatively long with 15 lamellae on fourth toe; and claws well developed.

Tail 37.8 mm in length, first 17.1 mm original, 6.1 mm in width at base, tapering to a point; dorsal scales of flat, square with conical, keeled tubercles, regenerated portion covered with small, smooth subcircular scales; median row of transversely expanded subcaudal scales, significantly larger than dorsal caudal scales on original portion; base of tail bearing hemipenal swellings; and postcloacal scales flat, imbricate.

#### Coloration in life

**(Fig. 10).** Ground color of dorsal head and dorsum dark grey with numerous small and irregular black blotches; lips with dark bars; two regularly disposed whitish tubercles on each side on each side running from postorbital along the flanks to tail, terminating at regenerated portion of tail; upper surface of fore and hind limbs uniformly dark brown with round black-brown spots; dorsum of tail covered with some large black-brown blotches; ventral surface beige uniformly as the belly and the throat.

#### Variation

**(Fig. 10).** The female paratype (VNUF R.2020.52) generally matches that of the holotype in all characteristics. The juvenile male paratype (NUOL R.2022.01) has fewer black blotches on head and dorsum and two regularly disposed whitish tubercles on each side on each side of the head extending from the postorbital region, along the flanks, to the tail tip. Further measurements are summarized in Tables 2–4 and Suppl. material 1: table S2.

#### Distribution.

*Dixoniusmuangfuangensis* sp. nov. currently is only known from the type locality of Nadan Village, Muangfuang District, Vientiane Province, Central Laos (Fig. 1).

#### Etymology.

The specific epithet of the new species refers to the type locality of the new species in Muangfuang District, Vientiane Province, Central Laos.

#### Natural history.

**Figure 11.**
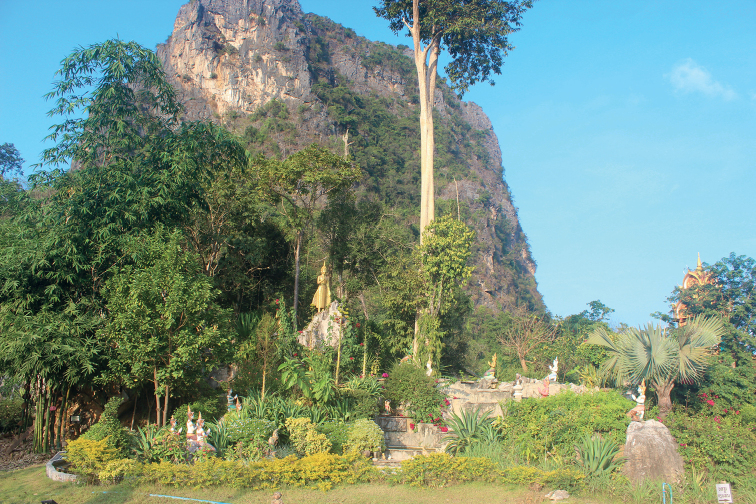
Habitat of *Dixoniusmuangfuangensis* sp. nov. Nadan Village, Muangfuang District, Vientiane Province, Central Laos.

The type series was collected between 19:10 and 19:30 h, on the ground inside Sinxay Temple, at an elevation of 276 m a.s.l. The surrounding habitat was disturbed lowland karst forest (Fig. 11).

#### Comparisons.

*Dixoniusmuangfuangensis* sp. nov. is the sister species to *D.lao* (Fig. 2) from which it differs by an uncorrected pairwise sequence divergence of 3.10% (Table 4). It is differentiated morphologically by having a significantly higher mean number of head length (HL), infralabials (IFL), and numbers of supralabial at midorbital position (MO). In addition, it differs from *D.lao* in dorsal pattern (dorsal pebble brown versus dorsal dark gray with black blotches). Statistically significant and discrete categorical differences between *Dixoniusmuangfuangensis* sp. nov. and all other species and populations are presented in Tables 5–7.

## ﻿Discussion

Morphological comparisons indicated that *Dixoniusgialaiensis* sp. nov. is most similar to its sister species *D.minhlei*, but can be differentiated from the latter species by the number of dorsal tubercle scale rows and differences in color pattern. The results of the molecular analysis show the uncorrected pairwise sequence divergence between the two taxa is 3.60%. Additionally, the two species are widely separated geographically being in different mountain systems and separated by the Dong Nai River system (Fig. 1). Collectively, these data suggest these are separate and distinct species.

*Dixoniusgialaiensis* sp. nov. was discovered in a protected forest near the National Highway 25. The construction of new infrastructure at this site strongly impacts the habitat of *D.gialaiensis* sp. nov., including range fragmentation and forest degradation. Further investigations on conservation status is urgently required to develop effective conservation measures.

*Dixoniusmuangfuangensis* sp. nov. is most closely related to *D.lao*, but can be distinguished from it by head shape and color pattern differences. The molecular analysis indicated these two species differ by a 3.1% uncorrected pairwise genetic distance. In addition, the two species evolved separately in geographically isolated regions. The type locality of *D.muangfuangensis* sp. nov. is approximately 500 km south of the type locality of *D.lao* and the type localities are separated by the Nam Ngiap and Xebangfai river network systems (Fig. 1).

The BEAST analysis indicates that the divergence between *Dixoniusgialaiensis* sp. nov. and *D.minhlei* and that between *D.muangfuangensis* sp. nov. and *D.lao* may have been the result of cyclical climatic events during the recent interglacial periods of the Pliocene as noted for several other Indochinese species (see Grismer and Grismer 2017 and references therein). *D.muangfuangensis* sp. nov. and *D.lao* diverged from one another at approximately 3.47 mya. Relatively soon after, at approximately 3.19 mya, *Dixoniusgialaiensis* sp. nov. and *D.minhlei* separated from one another, thus allowing sufficient time for them to evolve significant differences between them in a number of characteristics. During this time period, the formation of separate karstic habitats and granitic mountains and hills may have prevented gene flow between these populations, placing each species on separate evolutionary trajectories (Grismer and Grismer 2017).

## Supplementary Material

XML Treatment for Dixonius gialaiensis

XML Treatment for Dixonius muangfuangensis

## Supplementary materials

Supplementary material 1 Measurements and morphological characters of the type series of *Dixoniusgialaiensis* sp. nov. Data type tables (Excel spreadsheet) Explanation note table S1: Measurements (in mm) and morphological characters of the type series of *Dixoniusgialaiensis* sp. nov. (for abbreviations see Material and methods). Measurements taken on right side; FA is given in the left side; SPL/IFL/T4 given in right/ left order; -absence; * tail regenerated; table S2: Measurements (in mm) and morphological characters of the type series of *Dixoniusmuangfuangensis* sp. nov. (for abbreviations see material and methods). Measurements taken on right side; FA and T4 are given in the left side; SPL/IFL given in right/ left order; -absence, * tail regenerated; ** tail lost. https://binary.pensoft.net/file/853917This dataset is made available under the Open Database License (http://opendatacommons.org/licenses/odbl/1.0/). The Open Database License (ODbL) is a license agreement intended to allow users to freely share, modify, and use this Dataset while maintaining this same freedom for others, provided that the original source and author(s) are credited.Vinh Quang Luu, Thuong Huyen Nguyen, Minh Duc Le, Jesse L. Grismer, Hong Bich Ha, Saly Sitthivong, Tuoi Thi Hoang, L. Lee Grismer
